# Predicted molecular targets and pathways for germacrone, curdione, and furanodiene in the treatment of breast cancer using a bioinformatics approach

**DOI:** 10.1038/s41598-017-15812-9

**Published:** 2017-11-14

**Authors:** Qi Kong, Yong Ma, Jie Yu, Xiuping Chen

**Affiliations:** 1 Institute of Laboratory Animal Science, Chinese Academy of Medical Sciences (CAMS) and Comparative Medicine Center, Peking Union Medical College (PUMC); Key Laboratory of Human Disease Comparative Medicine, National Health and Family Planning Commission; Key Laboratory of Human Diseases Animal Model, State Administration of Traditional Chinese Medicine; Beijing Key Laboratory for Animal Models of Emerging and Remerging Infectious Diseases, Beijing, 100021 China; 2 Department of Urology, Shanxian Central Hospital, Heze, Shandong, 274300 China; 3 State Key Laboratory of Quality Research in Chinese Medicine Institute of Chinese Medical Sciences, University of Macau, Avenida da Universidade, Taipa, Macao, China

## Abstract

Germacrone, curdione, and furanodiene have been shown to be useful in the treatment of breast cancer but the pharmacological mechanism of action is unclear. In this paper, we explored a new method to study the molecular network and function of Traditional Chinese Medicine (TCM) herbs and their corresponding ingredients with bioinformatics tools, including PubChem Compound Database, BATMAN-TCM, SystemsDock, Coremine Medical, Gene ontology, and KEGG. Eleven targeted genes/proteins, 4 key pathways, and 10 biological processes were identified to participate in the mechanism of action in treating breast cancer with germacrone, curdione, and furanodiene. The information achieved by the bioinformatics tools was useful to interpretation the molecular mechanism for the treatment of germacrone, curdione, and furanodiene on breast cancers.

## Introduction

Traditional Chinese Medicine (TCM) has a rich history of thousands of years of clinical practice and plays a critical role in maintaining public health, especially in Asian countries. Especially, some compounds isolated from TCM such as artemisnin, arsenic trioxide, etc, have been successfully used in clinical practice for the treatments of malaria and APL respectively. Professor Youyou Tu, who firstly made the discovery of artemisnin, won the Nobel Prize in 2015. However, the molecular mechanisms behind TCM’s are often unclear, which has dramatically hindered international acceptance and popularity. TCM has been shown to target multiple signaling pathways in the therapy of cancers (such as cancers of the breast, prostate, colon, liver, lung, etc.), with low toxicity profiles compared to standard chemotherapeutic drugs^1,2^.

Breast cancer is a multi-factorial and multistep disease with high morbidity and mortality in women all over the world. Current therapies have limitations in their efficacy, especially in advanced cases^3,4^. In earlier studies, germacrone, curdione, and furanodiene were identified as three main ingredients in *Curcumae Wenyujin Y.H. Chenet C Ling*. Our previous studies showed that these three ingredients have anti-breast cancer effects and identified some potentially targeted proteins and genes^5^. However, the detailed molecular targets, mechanisms, and pathways involved remain to be elucidated.

Bioinformatics analysis methods, such as BATMAN-TCM (Bioinformatics Analysis Tool for Molecular mechANism of Traditional Chinese Medicine), SystemsDock, DAVID (the Database for Annotation, Visualization and Integrated Discovery), Swiss Target Prediction, KEGG (Kyoto Encyclopedia of Genes and Genomes), are commonly used to identify pathways and key genes or proteins in human diseases and to predict the potential effects of TCM ingredients^6^. Network pharmacology-based prediction of active ingredients and potential targets of TCM is increasing in popularity for TCM modernization and internationalization. In this paper, we identified the key genes/proteins and pathways for germacrone, curdione, and furanodiene in the treatment of breast cancer using bioinformatics analyses.

## Methods

**Figure 1:**
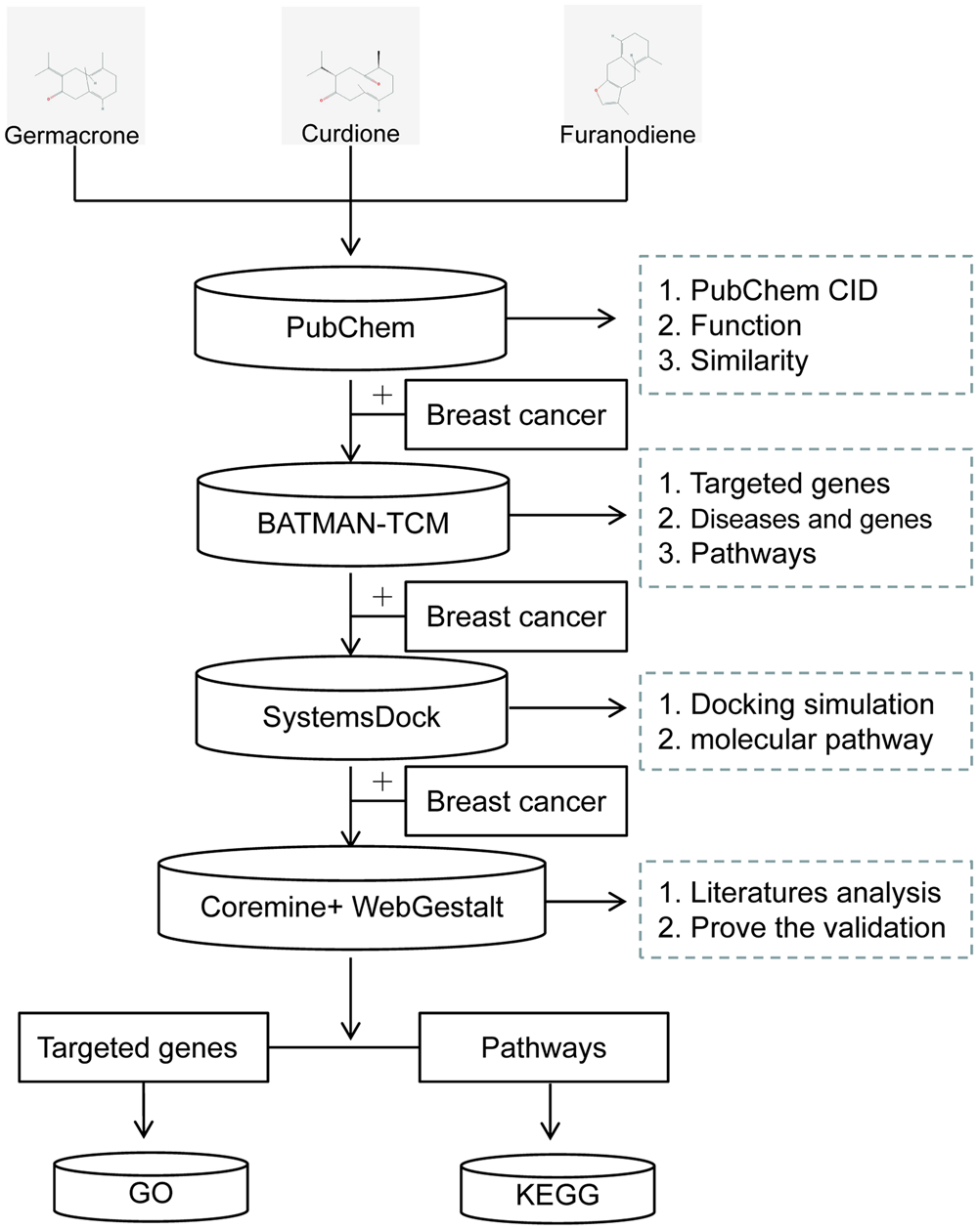
The flowchart of this whole analysis for this study.

A schematic of the entire analysis process is shown in Fig. 1. We first compared the biological characteristics of germacrone, curdione, and furanodiene using the PubChem Compound Database. We then used the PubChem_CID of germacrone, curdione, and furanodiene to analyze their function with BATMAN-TCM and identified potential genes/proteins and pathways involved. These targeted genes/proteins were further evaluated by SystemsDock with the high-precision docking simulation. The docked genes/proteins were validated by Coremine Medical, Network analyst, and a web-based gene set analysis toolkit based on scientific literature to assess their involvement in breast cancer. These targeted genes/proteins were also analyzed by Geneontology analysis and KEGG for further information regarding their functions and pathways. The information achieved by the above bioinformatics tools were useful to interpret the molecular mechanisms for germacrone, curdione, and furanodiene on breast cancers.

### Compare the biological characteristics of germacrone, curdione, and furanodiene

We compared the biological characteristics of germacrone, curdione, and furanodiene from the PubChem Compound database (https://www.ncbi.nlm.nih.gov/pccompound/). Some characteristics that were compared were structure, PubChem CID, CAS, Molecular Formula, Similarity, Function, Cellular Locations, etc.

### Functional analysis of TCM’s ingredients

BATMAN-TCM is the first tool for bioinformatics analysis for studying the molecular mechanism of TCM’s by predicting potential targets for ingredients in TCM’s, and then performing functional analyses on these targets including known drug/ingredient-target interactions, protein interaction networks, KEGG pathway data, etc. (http://bionet.ncpsb.org/BATMAN-TCM). The PubChem_CID/InChl of germacrone (6436348), curdione (6441391), and furanodiene (636458) were entered as search queries. The following parameter settings were used: Target Prediction’s Score cutoff was 20; Target Analysis’s Adjusted P-value is less than 0.05^7^.

### Network pharmacology-based prediction and analysis

SystemsDock (http://systemsdock.unit.oist.jp/) is a web server for network pharmacology-based prediction and analysis, which applies high-precision docking simulation and molecular pathway maps to comprehensively characterize a ligand’s selectivity and to illustrate how a ligand acts on a complex molecular network^8^. All the target prediction results of targeted genes/proteins from BATMAN-TCM for germacrone, curdione, and furanodiene were analyzed by SystemsDock to evaluate their relationship.

### Overview of the relationship between germacrone, curdione, and furanodiene with breast cancer

Coremine Medical (http://www.coremine.com/) is an ideal product for generating an overview of a complex subject while allowing for the possibility to narrow the scope of the subject to specific details ranging from introductory sources to the latest scientific literatures^9^. We used this tool to establish an overview of the complex relationship between germacrone, curdione, and furanodiene with breast cancer and found related information in health, medicine, and biology. The keywords of “germacrone”, “curdione”, “furanodiene”, and “breast cancer” were combined as inputs into the search field.

### Further analysis of key breast cancer genes by gene ontology and pathway enrichment

Geneontology analysis (GO, http://geneontology.org/) is a bioinformatics method for annotating genes and proteins and for identifying characteristic biological attributes for high-throughput genome data^10^. KEGG (http://www.genome.jp/) is a knowledge base for systematic analysis of gene functions, linking genomic information with higher-order functional information^11^. To analyze the targeted genes at a functional level, GO enrichment and KEGG pathway analysis were performed. P ≥ 0.05 was considered statistically significant. WebGestalt (WEB-based Gene SeT AnaLysis Toolket, http://www.webgestalt.org) is a functional enrichment analysis web tool to translate gene lists into biological insights.

### Verification of the scientific validity of targeted genes by PubMed and Clinical Trials

To identify the relationship between the 11 potential genes, the 3 ingredients of TCM, and breast cancer, we searched the PubMed (https://www.ncbi.nlm.nih.gov/pubmed) and Clinical Trials (https://clinicaltrials.gov) databases to find primary research and clinical trials, respectively, that have been carried out to validate the scientific relationships^12^.

### MTT assay

As previously described, cell viability was evaluated using the MTT assay^13^. Cells were treated with germacrone, curdione, furanodiene at various concentrations for 24 h. At the end of treatment, the cells were incubated with MTT (5 μg·mL 1) for 4 h at 37 °C. The formazan precipitate was dissolved with dimethyl sulfoxide. The absorbance at 570 nm was measured using a microplate reader (Molecular Devices, Sunnyvale, CA). The relative percentage of cells viability was expressed as percentage of that of the control cells.

### Western blotting

MCF7 cells were treated with germacrone, curdione and furanodiene at various concentrations and time. Cellular proteins were extracted from MCF7 cells in ice-cold lysis buffer containing 1% protein inhibitor cocktail and 1 mM phenylmethlsulfonyl fluoride (PMSF). The protein concentrations were quantified using the bicinchoninic acid (BCA) protein assay kit (Pierce Biotechnology). Thirty micrograms of the cellular proteins were separated by 8% SDS-PAGE and subsequently transferred to polyvinylidene difluoride (PVDF) membrane (Millipore, Bedfored, MA, USA). The membranes were blocked for 1 h at room temperature with 5% non-fat milk in a fresh TBS buffer containing 0.1% Tween-20 and then incubated with specific primary antibodies (1:1000) overnight at 4 °C. After incubation with the corresponding secondary antibodies (1:5000) for 1 h at room temperature, the reactive bands were identified using an enhanced chemiluminescence (ECL) detection reagent (Sigma, Sweden). The level of the loaded cellular proteins was normalized to the internal control GAPDH (glyceraldehyde-3-phosphate dehydrogenase).

### Validation by data mining tool analysis

The Kaplan Meier plotter (http://kmplot.com/) is capable to assess the effect of 54,675 genes on survival using 10,461 cancer samples. Primary purpose of the tool is a meta-analysis based biomarker assessment^14^.

## Results

### Compare the biological characteristics of germacrone, curdione, and furanodiene

We compared the biological characteristics and 2D structures of germacrone, curdione, and furanodiene by PubChem Compound database as shown in Table 1. The similarity score between curdione with germacrone is 0.85, while the similarity score between germacrone with furanodiene is 0.75. These results suggest that germacrone, curdione and furanodiene may have similar functions.

**Table 1:** Compare information of three tested integredients of TCM in Pubmed databases.

Compound Name	PubChem CID	CAS	Formula	Similarity	Functions
Germacrone	6436348	6902-91-6	C_15_H_22_O	1	Anti-androgenic cell cycle arrest and promoting apoptosis.
Curdione	6441391	13657-68-6	C_15_H_24_O_2_	0.85	Anti-neurocerebrovascular disorders.
Furanodiene	636458	—	C_15_H_20_O	0.75	Promoting apoptotic.

### Potential genes/proteins and pathways related to the mechanism of action of germacrone, curdione, and furanodiene

To predict the potential gene/proteins, pathways, and diseases germacrone, curdione, and furanodiene may be involved in or with, we tested the three ingredients in the BATMAN-TCM tool. Result 1 (target prediction results) of BATMAN-TCM by inputting the ingredients germacrone, curdione, and furanodiene are shown in Supplementary Table 1. There are 263 potential targets for germacrone and 264 potential targets for curdione, while there are no potential targets with scores larger than 20 for furanodiene. The potential targets for genes/proteins effected by germacrone and curdione were nearly the same with one different target, which was F12 (coagulation factor XII) for curdione. This result suggests that the structures and functions of germacrone and curdione may be similar to sesquiterpene lactone.

Result 2 (bioinformatics analyses of potential targets) shows the KEGG pathway, related disease, and gene ontology results. In the disease module, breast cancer is associated with 7 target genes/proteins (CYP19A1, ESR1, ESR2, HSP90AA1, PGR, SRC, VDR) as analyzed by TTD (Therapeutic Target Database, Supplementary Table 2)^15^ and 5 target genes/proteins (ATM, ESR1, PHB, PIK3CA, TP53) as analyzed by OMIM (Online Mendelian Inheritance in Man, Supplementary Table 3) that may be involved in the mechanism of germacrone and curdione treatment of breast cancer as the BATMAN-TCM predicted.Combining the two databases (OMIM and TTD), there are 11 unique target genes/proteins that may play a role in the mechanism of germacrone and curdione treatment of breast cancer, as shown in Table 2. Result 3 shows the results with significantly enriched KEGG pathways and OMIM/TTD disease phenotypes in a simplified network view (adjusted P-value < = 0.05).

**Table 2:** Potential genes/proteins for germacrone, curdione and furanodiene on breast cancer.

Databases	Diseases	Genes
TTD	Breast cancer	CYP19A1; ESR1; ESR2; HSP90AA1; PGR; SRC; VDR;
OMIM: 114480	Breast Cancer	ATM; ESR1; PHB; PIK3CA; TP53;
Combined TTD and OMIM	Breast Cancer	ATM; CYP19A1; ESR1; ESR2; HSP90AA1; PGR; PHB; PIK3CA; SRC; TP53; VDR;

Germacrone and curdione have been shown to have roles in signaling pathways involved in breast cancer, including the estrogen, MAPK, PI3K-Akt, Notch, Wnt, P53, and cell cycle signaling pathways, as listed in Supplementary Table 4 and marked with circles in Fig. 2. Germacrone and curdione are also involved in 132 other signaling pathways including purine metabolism, calcium, CGMP-PKG, neuroactive ligand-receptor interaction, cytokine-cytokine receptor interaction, etc. signaling pathways that could explain their broad effects on cancer, inflammation, cardiovascular disease, hypertension, and neurodegenerative diseases (Supplementary Tables 2, 3)^16^. Many of these signaling pathways were reported by the original papers in the molecular network of germacrone and curdione mechanism of treatment in breast cancer^17^.

**Figure 2:**
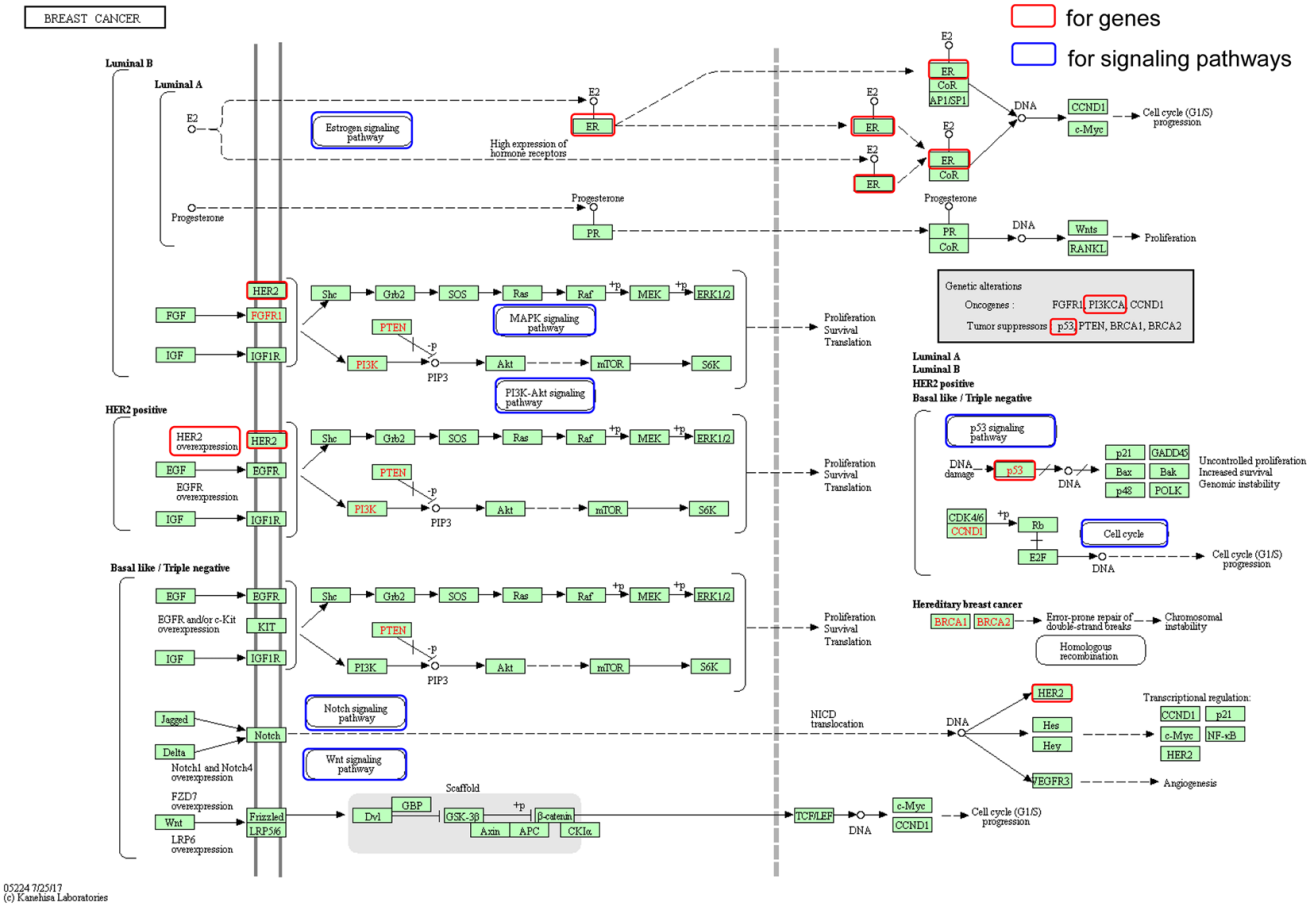
Signaling pathway of breast cancer download from KEGG website^33–35^ with targeted genes and pathways from germacrone, curdione, and furanodiene.

Analysis of the 11 genes/proteins identified for germacrone and curdione revealed 4 key signaling pathways from the KEGG pathway for breast cancer where 2 or more of the genes/proteins are involved. These 4 pathways are the estrogen (ESR1; ESR2; HSP90AA1; SRC), prolactin (ESR1; ESR2; SRC), thyroid hormone (ESR1; SRC), and progesterone-mediated oocyte maturation (HSP90AA1;PGR) signaling pathways^18^. These 4 key signaling pathways are all related to hormones, suggesting that germacrone and curdione may target hormones. Further evaluation of the mechanism needs to be done.

### Potential genes/proteins for breast cancer predicted from SystemsDock by docking simulation

**Figure 3:**
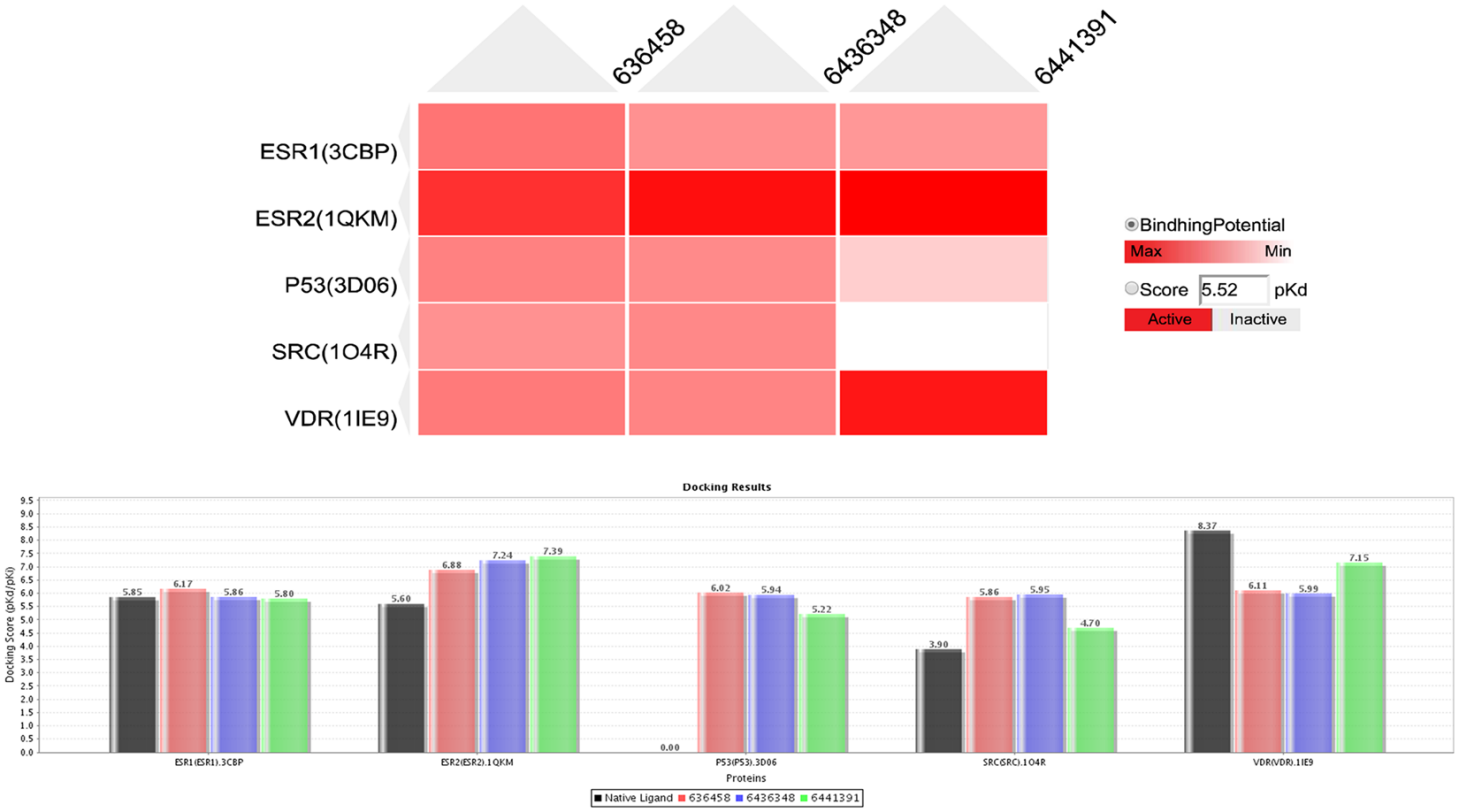
Results of the docking simulation in SystemsDock for Germacrone (6436348), Curdione (6441391), Furanodiene (636458) docking for 5 targeted genes/proteins. (**A**) Heat map of the docking simulation results. (**B**) Chart of the docking simulation results with the same result to A.

**Figure 4:**
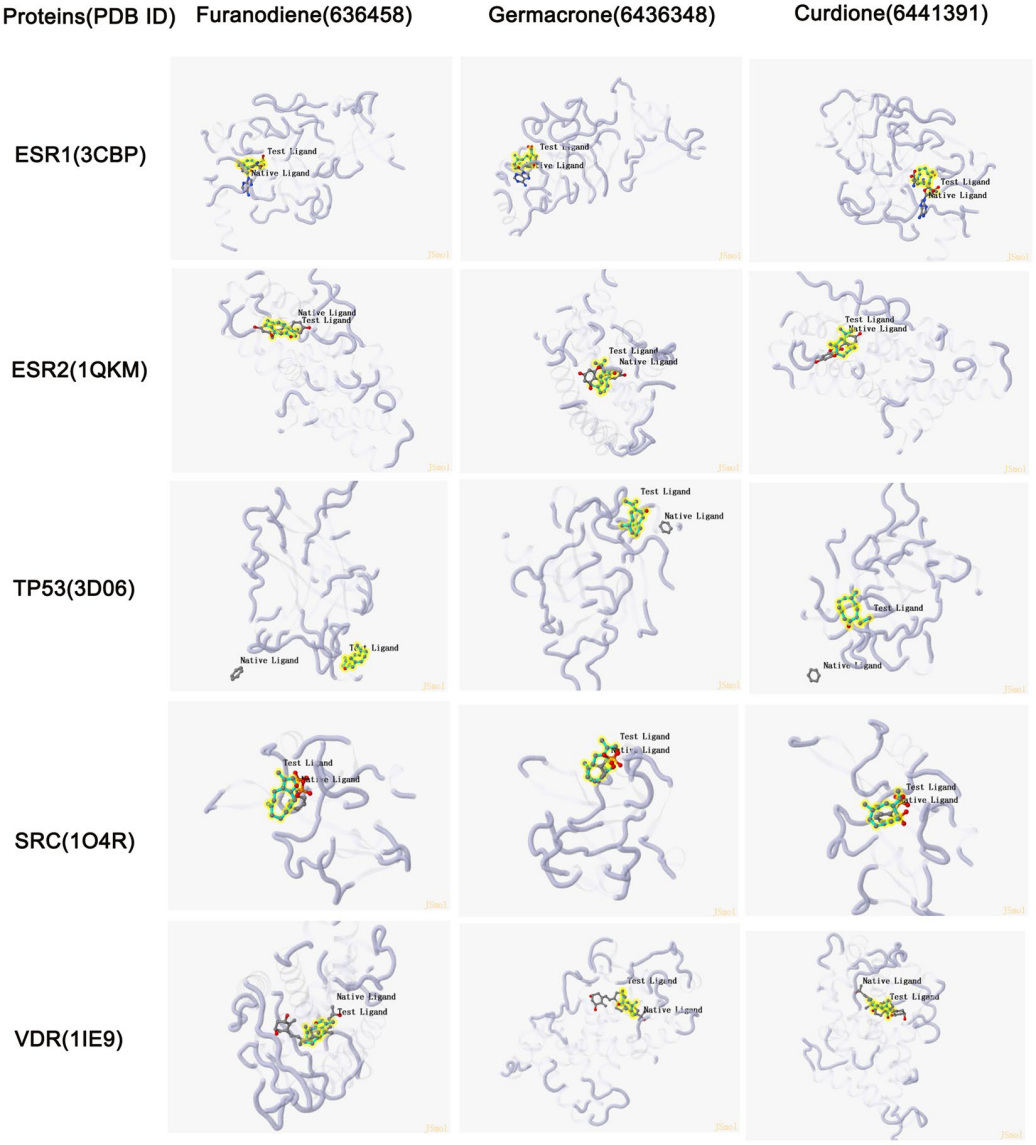
The detaild protein-ligand interactions of the docking simulation pose for each targeted proteins and intergradients of TCM.

The 11 potential targets (ATM, CYP19A1, ESR1, ESR2, HSP90AA1, PHB, PIK3CA, PGR, SRC, VDR, TP53) for germacrone and curdione were input into SystemsDock for analysis of the docking potential with germacrone, curdione, and furanodiene. The results show that 5 targets (ESR1, ESR2, TP53, SRC, and VDR) have protein structures that are compatible with germacrone, curdione, and furanodiene, while the other 6 targets’ binding sites have no specified proteins and were deleted from the list of positive targets. The docking scores (pKd/pKi) of the 5 positive targets are shown in Supplementary Table 5. From Fig. 3, ESR2 shows a higher docking score to germacrone, curdione, and furanodiene, especially for germacrone with a docking score of 7.376. VDR is also more sensitive to curdione with a docking score of 7.143. These results from the docking simulation of the 5 targets (ESR1, ESR2, TP53, SRC, VDR) highly suggests a role for the targets in the mechanism of germacrone, curdione, and furanodiene in treating breast cancer. The docking scores (pKd/pKi) of the docking simulation for each target protein and ingredient are shown in Table 3, while the detaild protein-ligand interactions of the docking simulation are shown in Fig. 4.

**Table 3:** The docking scores (pKd/pKi) of the docking simulation pose for each targeted proteins and intergradients of TCM.

Proteins(PDB ID)	Furanodiene(636458)	Germacrone(6436348)	Curdione(6441391)
ESR1(3CBP)	5.769	6.021	5.815
ESR2(1QKM)	6.883	7.349	7.376
P53(3D06)	6.02	5.94	5.209
SRC(1O4R)	5.866	5.951	4.69
VDR(1IE9)	6.157	5.96	7.143

### Complex relationship between germacrone, curdione, and furanodiene with breast cancer

In order to describe the relationship between germacrone, curdione, furanodiene, breast cancer, and the 5 targets (ESR1,ESR2,TP53,SRC,VDR) predicted from SystemsDock, we entered the above keywords into Coremine Medical to analyze the relationship. The 5 targets (ESR1, ESR2, TP53, SRC, VDR) have well established relationships with breast cancer but specific studies on their involvement in the effect of germacrone, curdione, and furanodiene on breast cancer is not well studied. Therefore, these results suggest that the targets identified in this study are novel targets in the mechanism of action of germacrone, curdione, and furanodiene in the treatment of breast cancer that need to be verified experimentally. There were 3 related genes/proteins (BCL2,CASP3,TNF) and 10 biological processes (cell proliferation, apoptosis, signal transduction, cell cycle, cell cycle arrest, growth, phosphorylation, signaling, G1 phase, and wound healing). BCL2 is commonly involved in pathways of cancers for TCM’s.

### Potential genes/proteins for germacrone, curdione, and furanodiene in breast cancer pathways

**Figure 5:**
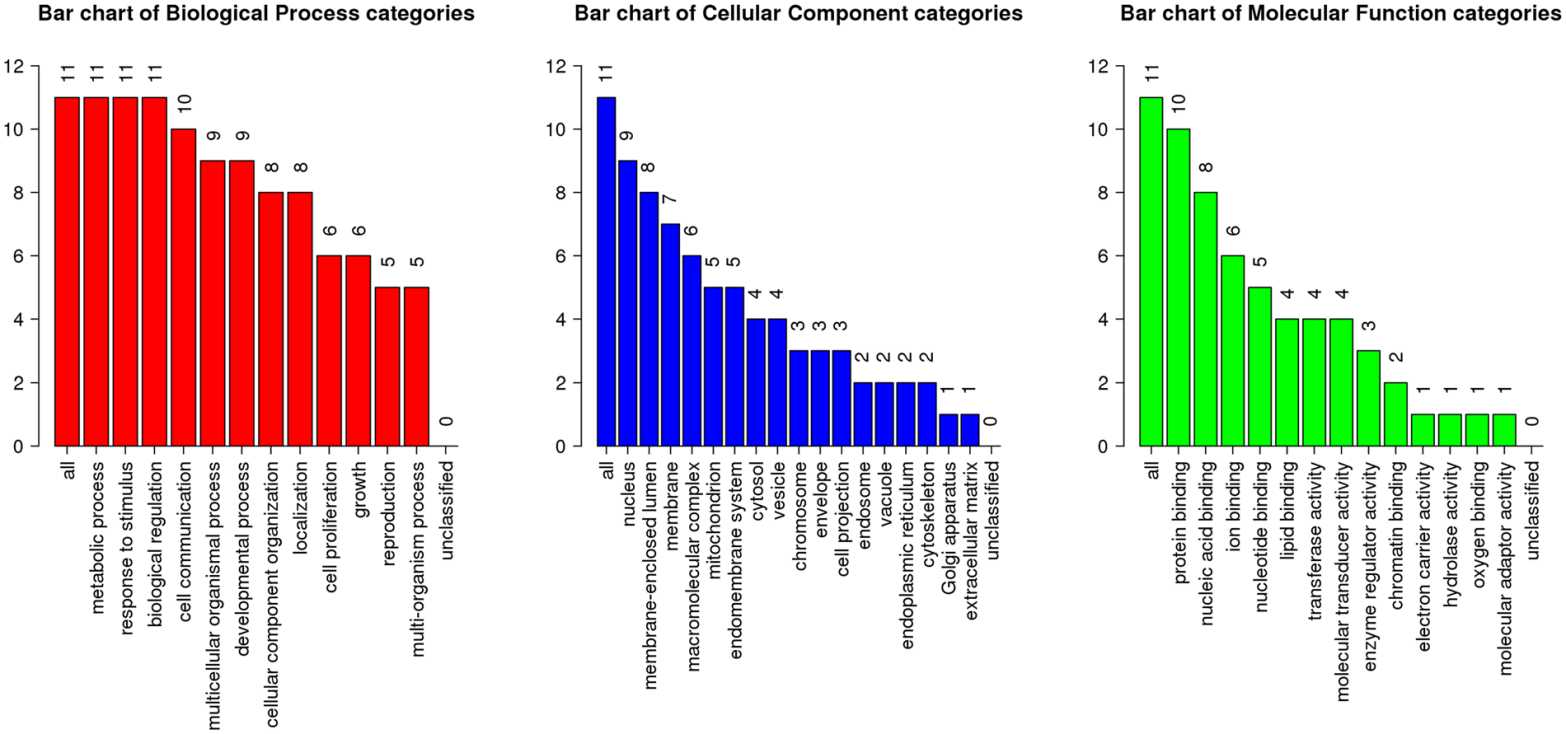
GOSlim summary for the user list genes. Each Biological Process, Cellular Component and Molecular Function category is represented by a red, blue and green bar, repectively. The height of the bar represents the number of user list genes observed in the category.

To study the role of the 5 targets (ESR1, ESR2, TP53, SRC, VDR) in the pathways of germacrone, curdione, and furanodiene in breast cancer, we searched KEGG and GO and the results are listed in Table 4. GO and KEGG pathway analysis further demonstrated these targets have relationships with breast cancer. We also evaluated the 11 target genes with WebGestalt and the GOSlim summary is shown in Fig. 5. Overrepresentation Enrichment Analysis (ORA) results of the results obtained from DisGeNET showed mammary neoplasm, adenocarcinoma, prostatic neoplasm, bladder neoplasm, and liver carcinoma were enriched categories for the 11 targets, suggesting that germacrone, curdione, and furanodiene may have roles in the treatment of these diseases (Supplementary Table 6).

**Table 4:** The combined information for the 5 potential target genes/proteins that germacrone, curdione on breast cancer.

Genes ID	Other Name	Common name	Scores in BATMAN-TCM	KEGG Pathway	Entrez ID	GO Functions
ESR1	ER; ESR; ESRA; ESTRR; Era; NR3A1	estrogen receptor 1	80.882	Estrogen signaling pathway;Prolactin Signaling Pathway	2099	Involved in pathological processes including breast cancer, endometrial cancer, and osteoporosis.
ESR2	ER-BETA, ESR-BETA, ESRB, ESTRB, Erb, NR3A2	estrogen receptor 2	22.373	Estrogen Signaling Pathway;Prolactin Signaling Pathway	2100	The gene product contains an N-terminal DNA binding domain and C-terminal ligand binding domain and is localized to the nucleus, cytoplasm, and mitochondria.
SRC	ASV; SRC1; THC6; c-SRC; p60-Src	tyrosine-protein kinase Src	22.373	Estrogen Signaling Pathway;Prolactin Signaling Pathway;Oxytocin Signaling Pathway;	6714	May play a role in the regulation of embryonic development and cell growth.
TP53	BCC7; LFS1; P53; TRP53	tumor protein p53	22.373	P53 Signaling Pathway	7157	Mutations in this gene are associated with a variety of human cancers
VDR	NR1I1; PPP1R163	vitamin D (1,25- dihydroxyvitamin D3) receptor	22.373	Endocrine And Other Factor-Regulated Calcium Reabsorption;Mineral Absorption	7421	Principally involved in mineral metabolism though the receptor regulates a variety of other metabolic pathways, such as those involved in the immune response and cancer.

Among the 11 genes/proteins, all the genes had had a role in metabolic processes, responses to stimulus, and infer the biological regulation, cell communication etc. With respect to the cellular compartmentalization, 9 genes are located in the nucleus, 8 genes are located in membrane-enclosed lumens. 10 genes/proteins have functions in protein binding, 8 genes/proteins can bind nucleic acids, 4 genes/proteins have transferase, molecular transducer, and enzyme regulator activities. Supplementary Table 7 shows the results of scientific validation of targeted genes studied in breast cancer by PubMed and Clinical Trials.

### MTT assay and Western blotting results for targeted genes/proteins

**Figure 6:**
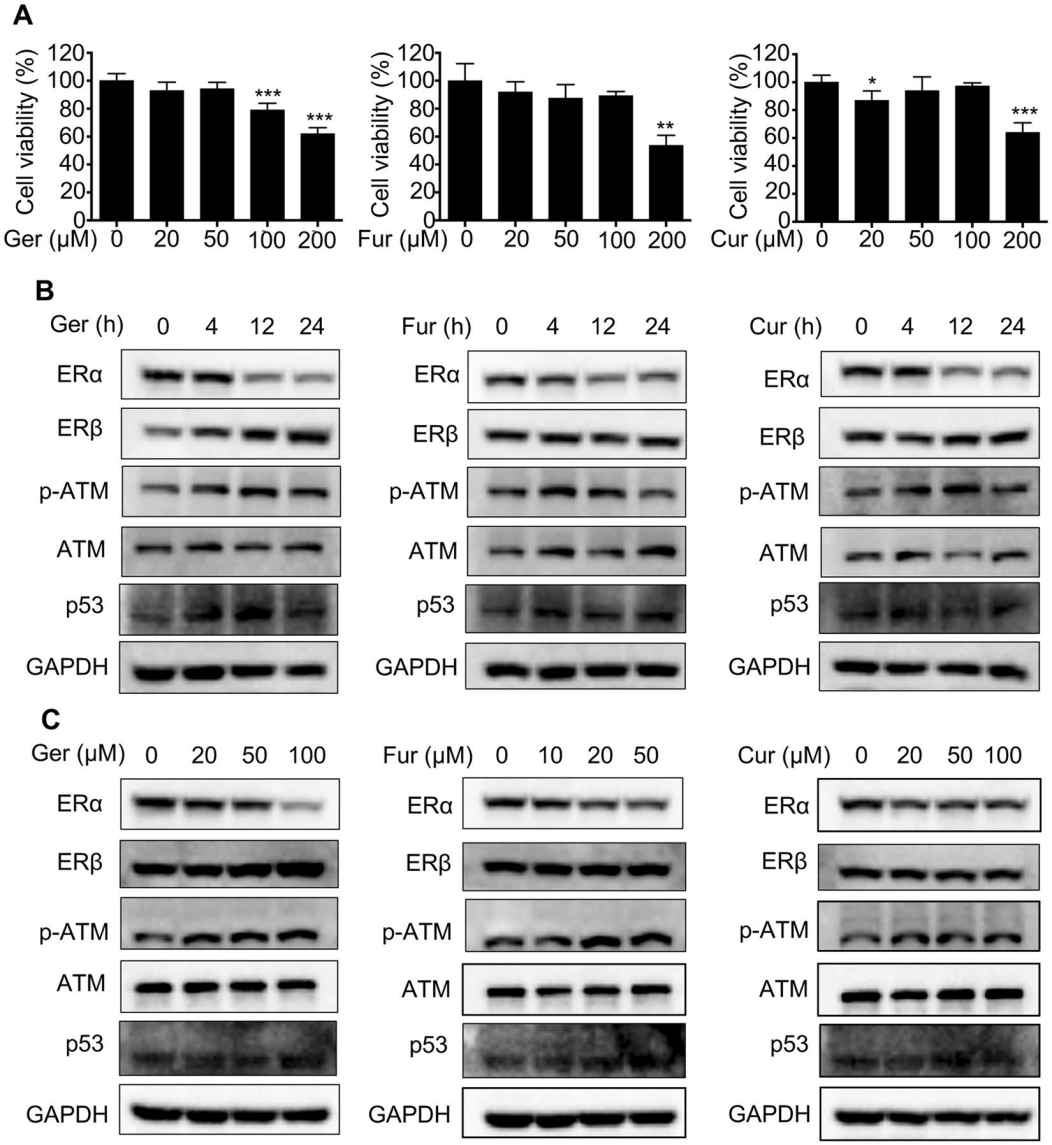
Results of MTT assay and Western blotting test. (**A**) The cytotoxic effect of germacrone, curdione, and furanodiene on MCF7 breast cancer cells. Cells were treated with the indicated concentrations of germacrone, curdione, and furanodiene for 24 h and MTT assay was performed. Data represent the mean ± SEM (n = 3). *P < 0.05, **P < 0.01, ***P < 0.001 for each group versus control. (**B**,**C**) Germacrone, curdione, and furanodiene regulated the target protein level. Cells were treated with germacrone, curdione, and furanodiene for various time or concentrations, and the protein expression was determined by western blotting. ERα and ERβ are encoded by the ESR1 and ESR2 gene seperately.

To investigated the cytotoxicity of germacrone, curdione and furanodiene in MCF7 cells, we treated the cells with germacrone (20, 50, 100 and 200 µM), curdione (20, 50, 100 and 200 µM), furanodiene (20, 50, 100 and 200 µM) at various concentrations for 24 h. After treatment, the cell viability was evaluated by MTT assay. Compared with the control group, 200 uM germacrone and curdione, 100 uM furanodiene significantly inhibit cell viability (Fig. 6A).

In this study, germacrone, curdione, and furanodiene are shown to potentially target breast cancer through some genes, such as ESR1, ESR2, p-ATM, ATM and TP53. To confirm the effect of germacrone, curdione, and furanodiene in the treatment of breast cancers, we detect the protein level using western blotting in MCF7 cells. We found that both germacrone, curdione, and furanodiene down-regulated the ESR1 protein expression, and little effect on regulating the ESR2 protein expression in ESR1-positive MCF-7 cells (Fig. 6B,C), while up-regulated p-ATM and p53 protein expression, which was consistence with the previously reported^19,20^. Moreover, it further confirmed our results in this study. Full-length gels/blots in Fig. 6B,C are presented in the Supplementary Figure 1.

### Annalysis results from the Kaplan Meier plotter tool

**Figure 7:**
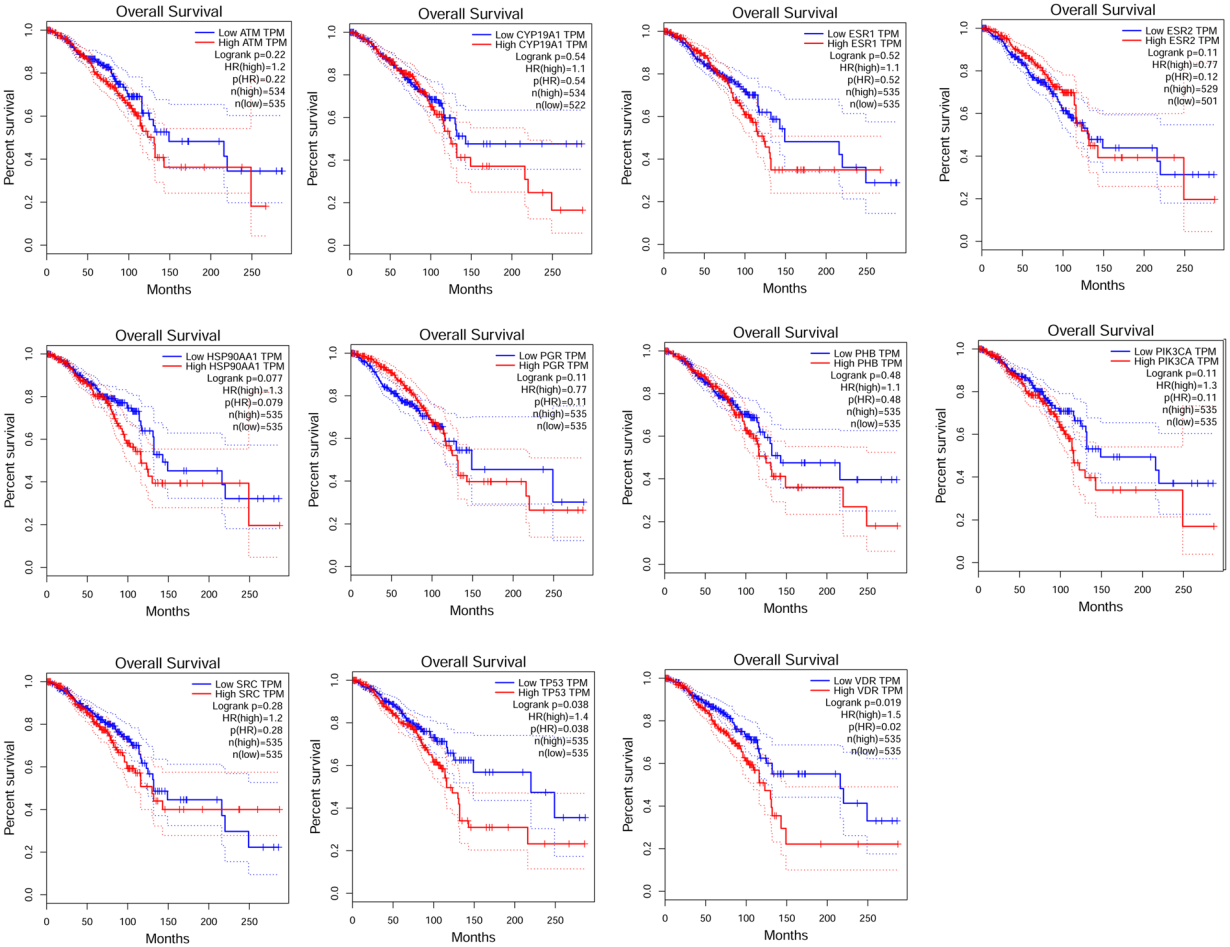
Kaplan–Meier survival curves show significant overall survival (OS) or disease-free survival (DFS) time differences of 11 targed genes between TP53-mutated and TP53-wildtype cancer patients (log-rank test, unadjusted P-value < 0.05).

Survival analysis sourced from TCGA data was performed and the result showed that these targeted genes were presented to be up-regulated or down-regulated in different cancer types, and Kaplan-Meier analysis curves demonstrated that aberrantly expression of both miRs was conspicuously associated with poor overall survival (Fig. 7). We also analysised the 11 targeted genes expression levels in MCF7 cell using the human protein atlas (Supplementary Figure 2).

## Discussion

The rapidly advancing research on traditional Chinese medicine (TCM) has greatly increased interest from pharmaceutical industries worldwide. Complex sesquiterpenoids from the leaves of Chloranthus tianmushanensis have tyrosine kinase inhibitory activity^21^. *Curcuma phaeocaulis Valeton* is a commonly prescribed Chinese medical herb for cancer therapy. The main ingredients of the extract (Cpv) were identified to include germacrone, curdione, and furanodiene. Cpv treatment significantly inhibited cell proliferation, increased LDH release, and induced intracellular ROS formation. Cpv increased the protein expression of Bax, PARP, cleaved PARP, caspase-3 and 7, JNK1, p-p42/44 MAPK, NF-κB, IKKα, IKKβ and decreased the protein expression of Bcl-2, Bcl-xL, Bim, Bik, Bad, integrin β5, p42/44 MAPK without affecting integrin α5, β1, and p38 MAPK protein expression^22,23^.

As an alternative treatment for breast cancer, ingredients isolated from natural products have been extensively investigated. In our previous study, we showed that germacrone inhibits the proliferation and migration of MCF-7 breast cancer cells^5^ by the inhibition of ERα signaling^24^. The expressions of the anti-apoptotic Bcl-2 and Bcl-xL protein and the pro-apoptotic TP53 and TP21 were dose-dependently regulated by germacrone as well^25–27^. While the research on curdione and furanodiene are limited, the results from this study provide a good starting point for further studies.

Li J, *et al*. reported that curdione significantly suppressed tumor growth in a MCF-7 xenograft nude mouse breast tumor model in a dose-dependent manner. The expression of apoptosis-related proteins including cleaved caspase-3, caspase-9 and Bax was increased in curdione treatment groups, while the expression of the anti-apoptotic Bcl-2 was decreased^28^. Sun XY *et al*. reported that *in vivo*, furanodiene was also found to exhibit inhibitory effects on the growth of uterine cervical (U14) and sarcoma 180 (Sl80) tumors in mice. Compared to the essential oil, furanodiene showed stronger growth inhibitions on Hela, Hep-2, HL-60, PC3, SGC-7901 and HT-1080 cells with IC(50) between 0.6–4.8 microg/ml^29^. Zhong, Z. *et al*. reportd germacrone inhibits estrogen-dependent proliferation and target gene expression. Germacrone treatment significantly reduced the mRNA level of TFF1, GREB1, and PGR^30^. These published papers further confirmed our results in this study.

In this study, germacrone, curdione, and furanodiene are shown to potentially target breast cancer through 11 genes, especially ESR1, ESR2, TP53, SRC, and VDR. Several signaling pathways were involved in the molecular machnism for these effect, including estrogen, MAPK, PI3K-Akt, Notch, Wnt, P53, and cell cycle signaling pathways (Fig. 2). Among which, ESR2 was shown to be involved in the estrogen and prolactin signaling pathways and may take a significant role in the effect of germacrone, curdione, and furanodiene in the treatment of breast cancers and should be studied in animal models. Results from SystemsDock showed that the P53 and SRC proteins have lower docking scores than ESR2, ESR1, and VDR with germacrone and curdione, while the data showed they have enough protein-ligand interactions of the docking pose (Fig. 3).

Germacrone, curdione, and furanodiene are also involved in the treatment of other human diseases, such as prostate cancer, osteosarcoma, ovarian cancer, inflammation, asthma, cardiovascular disease, hypertension, analgesics, neurodegenerative diseases, malaria, human immunodeficiency virus type 1 (HIV-1), diabetes mellitus, and these results are confirmed by previous research and clinical trials (Supplementary Table 7). Our results showed that the effect of germacrone, curdione, and furanodiene on breast cancer may involve 10 biological processes (cell proliferation, apoptotic process, signal transduction, cell cycle, cell cycle arrest, growth, phosphorylation, signaling, G1 phase, wound healing), which have also been reported by primary research papers. These results identify potential future research emphasis on the mechanism for germacrone, curdione, and furanodiene on human disease and will ultimately promote their application in clinical practice.

As reported, Artemisinin, triptolide, celastrol, capsaicin, and curcumin are “poster children” for the power and promise of converting traditional medicines into modern drugs^31^. Curcumin is extracted from the same curcuma herbs as germacrone, curdione, and furanodiene, suggesting they may have similar functions^32^. Germacrone, curdione, and furanodiene shows both an anti-cancer and anti-inflammatory effect. Their effect on inflammation may also play a role in anti-cancer treatment, which also needs to be further studied.

## Electronic supplementary material

Supplementary materials
