# Supplementary material for: Predicted molecular targets and pathways for germacrone, curdione, and furanodiene in the treatment of breast cancer using a bioinformatics approach
Source: Sci Rep. 2017 Nov 14;7:15543. doi: 10.1038/s41598-017-15812-9 (PMC5686110; doi:10.1038/s41598-017-15812-9)
Supplement: Supplementary file 1 — Supplementary materials [file 41598_2017_15812_MOESM1_ESM.doc]

**Predicted molecular targets and pathways for germacrone, curdione, and furanodiene in the treatment of breast cancer using a bioinformatics approach**

**Qi Kong**^1,*^**, Yong Ma**^2^**,, Jie Yu**^3^**, and Xiuping Chen**^3^

^1^ Institute of Laboratory Animal Science, Chinese Academy of Medical Sciences (CAMS) and Comparative Medicine Center, Peking Union Medical College (PUMC); Key Laboratory of Human Disease Comparative Medicine, Ministry of Health; Key Laboratory of Human Diseases Animal Model,State Administration of Traditional Chinese Medicine; Beijing Key Laboratory for Animal Models of Emerging and Remerging Infectious Diseases, Beijing, 100021, China.

^2^ Urology Surgery, Shanxian Central Hospital, Shandong, 274300,China.

^3^ State Key Laboratory of Quality Research in Chinese Medicine, Institute of Chinese Medical Sciences, University of Macau, Macau, China.

^*^corresponding to: latc281@163.com.

**Supplementary materials**

**Figure 6 B**

**
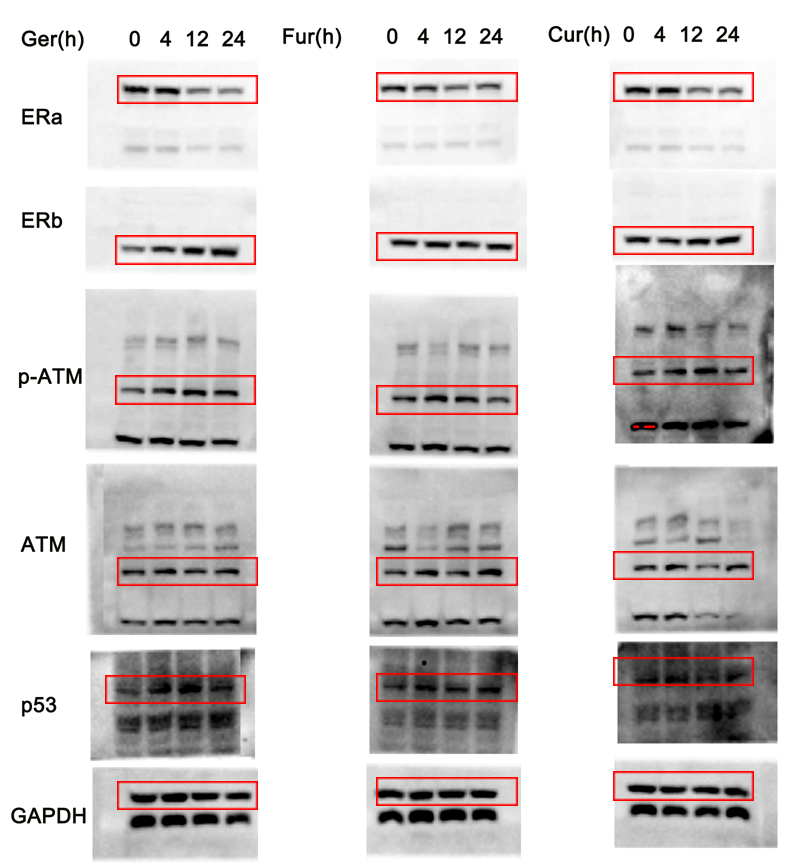
**

**Figure 6 C**

**
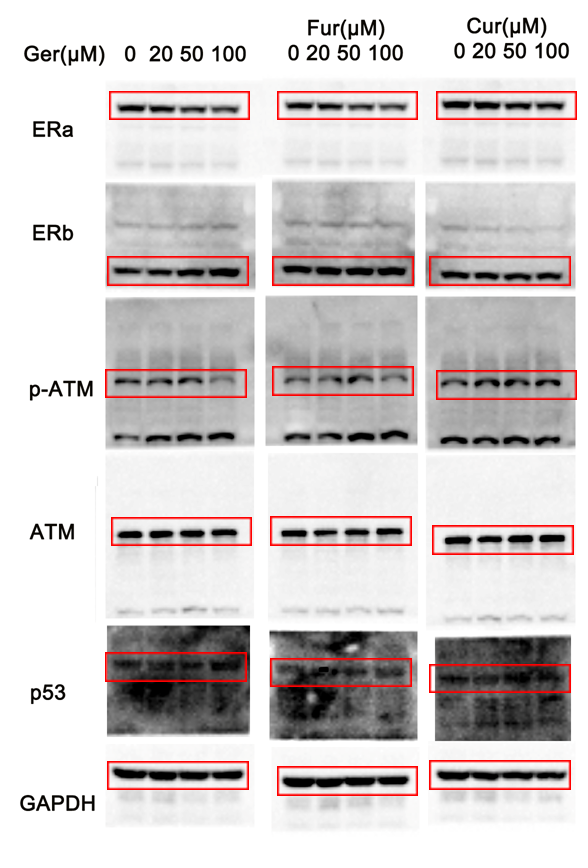
**

**Supplementary figure 1 (according to Figure 6 B, C)** Germacrone, curdione, and furanodiene regulated the target protein level. Cells were treated with germacrone, curdione, and furanodiene for various time or concentrations, and the protein expression was determined by western blotting. ERα and ERβ are encoded by the ESR1 and ESR2 gene seperately. The cirled figures in red are these where cropped gels/blots are displayed in Figure 6 B, C.

**Supplementary figure 2**. 11 targeted genes expression levels in MCF7_BREAST cell using the human protein atlas.

**Supplementary table 1. Results from BATMAN-TCM by input ingredients of germacrone, curdione and furanodiene for analysis.**

| **Compound** | **Predicted targets [Gene Symbol] ranked according to the decreasing ([score](http://bionet.ncpsb.org/batman-tcm/index.php/Home/document/index#Algorithm))** |
| --- | --- |
| germacrone | [PDE7B](http://www.ncbi.nlm.nih.gov/gene/?term=27115)(80.882)[PDE5A](http://www.ncbi.nlm.nih.gov/gene/?term=8654)(80.882)[PDE9A](http://www.ncbi.nlm.nih.gov/gene/?term=5152)(80.882)[PDE4A](http://www.ncbi.nlm.nih.gov/gene/?term=5141)(80.882)[RYR1](http://www.ncbi.nlm.nih.gov/gene/?term=6261)(80.882) [CYP17A1](http://www.ncbi.nlm.nih.gov/gene/?term=1586)(80.882)[PDE3A](http://www.ncbi.nlm.nih.gov/gene/?term=5139)(80.882)[ADORA2A](http://www.ncbi.nlm.nih.gov/gene/?term=135)(80.882)[ITPR1](http://www.ncbi.nlm.nih.gov/gene/?term=3708)(80.882)[PDE6A](http://www.ncbi.nlm.nih.gov/gene/?term=5145)(80.882) [PRKDC](http://www.ncbi.nlm.nih.gov/gene/?term=5591)(80.882)[PIK3CD](http://www.ncbi.nlm.nih.gov/gene/?term=5293)(80.882)[ESR1](http://www.ncbi.nlm.nih.gov/gene/?term=2099)(80.882)[PDE3B](http://www.ncbi.nlm.nih.gov/gene/?term=5140)(80.882)[PGD](http://www.ncbi.nlm.nih.gov/gene/?term=5226)(80.882) [PIK3CA](http://www.ncbi.nlm.nih.gov/gene/?term=5290)(80.882)[ADORA2B](http://www.ncbi.nlm.nih.gov/gene/?term=136)(80.882)[PDE1A](http://www.ncbi.nlm.nih.gov/gene/?term=5136)(80.882)[PDE4D](http://www.ncbi.nlm.nih.gov/gene/?term=5144)(80.882)[PGR](http://www.ncbi.nlm.nih.gov/gene/?term=5241)(80.882) [PIK3CB](http://www.ncbi.nlm.nih.gov/gene/?term=5291)(80.882)[ITPR2](http://www.ncbi.nlm.nih.gov/gene/?term=3709)(80.882)[PDE1B](http://www.ncbi.nlm.nih.gov/gene/?term=5153)(80.882)[OPRK1](http://www.ncbi.nlm.nih.gov/gene/?term=4986)(80.882)[PDE7A](http://www.ncbi.nlm.nih.gov/gene/?term=5150)(80.882) [ITPR3](http://www.ncbi.nlm.nih.gov/gene/?term=3710)(80.882)[POLA2](http://www.ncbi.nlm.nih.gov/gene/?term=23649)(80.882)[PDE4C](http://www.ncbi.nlm.nih.gov/gene/?term=5143)(80.882)[PDE10A](http://www.ncbi.nlm.nih.gov/gene/?term=10846)(80.882)[PDE1C](http://www.ncbi.nlm.nih.gov/gene/?term=5137)(80.882) [ATM](http://www.ncbi.nlm.nih.gov/gene/?term=472)(80.882)[PDE6B](http://www.ncbi.nlm.nih.gov/gene/?term=5158)(80.882)[PDE4B](http://www.ncbi.nlm.nih.gov/gene/?term=5142)(80.882)[PDE2A](http://www.ncbi.nlm.nih.gov/gene/?term=5138)(80.882)[ADORA1](http://www.ncbi.nlm.nih.gov/gene/?term=134)(80.882) [CYP19A1](http://www.ncbi.nlm.nih.gov/gene/?term=1588)(80.882)[PDE8B](http://www.ncbi.nlm.nih.gov/gene/?term=8622)(80.882)[NT5E](http://www.ncbi.nlm.nih.gov/gene/?term=4907)(80.882)[HDAC2](http://www.ncbi.nlm.nih.gov/gene/?term=3066)(80.882)[PDE6C](http://www.ncbi.nlm.nih.gov/gene/?term=5146)(80.882) [PDE11A](http://www.ncbi.nlm.nih.gov/gene/?term=50940)(80.882)[PDE8A](http://www.ncbi.nlm.nih.gov/gene/?term=5151)(80.882)[NR3C2](http://www.ncbi.nlm.nih.gov/gene/?term=4306)(80.882)[RINT1](http://www.ncbi.nlm.nih.gov/gene/?term=60561)(55.444)[RIPK1](http://www.ncbi.nlm.nih.gov/gene/?term=8737)(55.444) [PIK3R1](http://www.ncbi.nlm.nih.gov/gene/?term=5295)(55.444)[WNT4](http://www.ncbi.nlm.nih.gov/gene/?term=54361)(55.444)[TACR2](http://www.ncbi.nlm.nih.gov/gene/?term=6865)(55.444)[AMPD3](http://www.ncbi.nlm.nih.gov/gene/?term=272)(55.444)[CX3CR1](http://www.ncbi.nlm.nih.gov/gene/?term=1524)(55.444) [ADA](http://www.ncbi.nlm.nih.gov/gene/?term=100)(55.444)[HAP1](http://www.ncbi.nlm.nih.gov/gene/?term=9001)(55.444)[IDNK](http://www.ncbi.nlm.nih.gov/gene/?term=414328)(55.444)[HDAC1](http://www.ncbi.nlm.nih.gov/gene/?term=3065)(23.000)[TFAP2C](http://www.ncbi.nlm.nih.gov/gene/?term=7022)(22.373) [HMGA2](http://www.ncbi.nlm.nih.gov/gene/?term=8091)(22.373)[TNFSF11](http://www.ncbi.nlm.nih.gov/gene/?term=8600)(22.373)[NT5C1A](http://www.ncbi.nlm.nih.gov/gene/?term=84618)(22.373)[ACTN3](http://www.ncbi.nlm.nih.gov/gene/?term=89)(22.373)[NOS1AP](http://www.ncbi.nlm.nih.gov/gene/?term=9722)(22.373) [TAC1](http://www.ncbi.nlm.nih.gov/gene/?term=6863)(22.373)[PHB](http://www.ncbi.nlm.nih.gov/gene/?term=5245)(22.373)[SIRT1](http://www.ncbi.nlm.nih.gov/gene/?term=23411)(22.373)[DGKI](http://www.ncbi.nlm.nih.gov/gene/?term=9162)(22.373)[FKBP1A](http://www.ncbi.nlm.nih.gov/gene/?term=2280)(22.373) [FURIN](http://www.ncbi.nlm.nih.gov/gene/?term=5045)(22.373)[TOX3](http://www.ncbi.nlm.nih.gov/gene/?term=27324)(22.373)[UTS2](http://www.ncbi.nlm.nih.gov/gene/?term=10911)(22.373)[STUB1](http://www.ncbi.nlm.nih.gov/gene/?term=10273)(22.373)[CTR9](http://www.ncbi.nlm.nih.gov/gene/?term=9646)(22.373) [G6PD](http://www.ncbi.nlm.nih.gov/gene/?term=2539)(22.373)[GNAT1](http://www.ncbi.nlm.nih.gov/gene/?term=2779)(22.373)[SIX1](http://www.ncbi.nlm.nih.gov/gene/?term=6495)(22.373)[QDPR](http://www.ncbi.nlm.nih.gov/gene/?term=5860)(22.373)[TGFB1](http://www.ncbi.nlm.nih.gov/gene/?term=7040)(22.373) [ATP11C](http://www.ncbi.nlm.nih.gov/gene/?term=286410)(22.373)[KHDRBS1](http://www.ncbi.nlm.nih.gov/gene/?term=10657)(22.373)[PPP3CB](http://www.ncbi.nlm.nih.gov/gene/?term=5532)(22.373)[SYT2](http://www.ncbi.nlm.nih.gov/gene/?term=127833)(22.373)[SIX4](http://www.ncbi.nlm.nih.gov/gene/?term=51804)(22.373) [SIRT2](http://www.ncbi.nlm.nih.gov/gene/?term=22933)(22.373)[SPI1](http://www.ncbi.nlm.nih.gov/gene/?term=6688)(22.373)[HOMER1](http://www.ncbi.nlm.nih.gov/gene/?term=9456)(22.373)[SHANK3](http://www.ncbi.nlm.nih.gov/gene/?term=85358)(22.373)[TAS1R1](http://www.ncbi.nlm.nih.gov/gene/?term=80835)(22.373) [TRIM28](http://www.ncbi.nlm.nih.gov/gene/?term=10155)(22.373)[XRCC4](http://www.ncbi.nlm.nih.gov/gene/?term=7518)(22.373)[MED1](http://www.ncbi.nlm.nih.gov/gene/?term=5469)(22.373)[NKX3-1](http://www.ncbi.nlm.nih.gov/gene/?term=4824)(22.373)[UBE2B](http://www.ncbi.nlm.nih.gov/gene/?term=7320)(22.373) [ESR2](http://www.ncbi.nlm.nih.gov/gene/?term=2100)(22.373)[HCN4](http://www.ncbi.nlm.nih.gov/gene/?term=10021)(22.373)[EDN1](http://www.ncbi.nlm.nih.gov/gene/?term=1906)(22.373)[CACNA1A](http://www.ncbi.nlm.nih.gov/gene/?term=773)(22.373)[STAT5A](http://www.ncbi.nlm.nih.gov/gene/?term=6776)(22.373) [TP53](http://www.ncbi.nlm.nih.gov/gene/?term=7157)(22.373)[GNAT3](http://www.ncbi.nlm.nih.gov/gene/?term=346562)(22.373)[HELB](http://www.ncbi.nlm.nih.gov/gene/?term=92797)(22.373)[COMT](http://www.ncbi.nlm.nih.gov/gene/?term=1312)(22.373)[SLC26A6](http://www.ncbi.nlm.nih.gov/gene/?term=65010)(22.373) [ADRA2A](http://www.ncbi.nlm.nih.gov/gene/?term=150)(22.373)[YWHAE](http://www.ncbi.nlm.nih.gov/gene/?term=7531)(22.373)[CETN2](http://www.ncbi.nlm.nih.gov/gene/?term=1069)(22.373)[POLA1](http://www.ncbi.nlm.nih.gov/gene/?term=5422)(22.373)[CRP](http://www.ncbi.nlm.nih.gov/gene/?term=1401)(22.373) [CETN1](http://www.ncbi.nlm.nih.gov/gene/?term=1068)(22.373)[NUDT9](http://www.ncbi.nlm.nih.gov/gene/?term=53343)(22.373)[PIK3CG](http://www.ncbi.nlm.nih.gov/gene/?term=5294)(22.373)[BMP2](http://www.ncbi.nlm.nih.gov/gene/?term=650)(22.373)[TNF](http://www.ncbi.nlm.nih.gov/gene/?term=7124)(22.373) [NEDD4](http://www.ncbi.nlm.nih.gov/gene/?term=4734)(22.373)[AGT](http://www.ncbi.nlm.nih.gov/gene/?term=183)(22.373)[FER](http://www.ncbi.nlm.nih.gov/gene/?term=2241)(22.373)[SHH](http://www.ncbi.nlm.nih.gov/gene/?term=6469)(22.373)[SERPINB3](http://www.ncbi.nlm.nih.gov/gene/?term=6317)(22.373) [FOXP3](http://www.ncbi.nlm.nih.gov/gene/?term=50943)(22.373)[CXCL13](http://www.ncbi.nlm.nih.gov/gene/?term=10563)(22.373)[ARID1A](http://www.ncbi.nlm.nih.gov/gene/?term=8289)(22.373)[FGF10](http://www.ncbi.nlm.nih.gov/gene/?term=2255)(22.373)[KCNB1](http://www.ncbi.nlm.nih.gov/gene/?term=3745)(22.373) [LEF1](http://www.ncbi.nlm.nih.gov/gene/?term=51176)(22.373)[PTK2B](http://www.ncbi.nlm.nih.gov/gene/?term=2185)(22.373)[LIG4](http://www.ncbi.nlm.nih.gov/gene/?term=3981)(22.373)[ADRBK1](http://www.ncbi.nlm.nih.gov/gene/?term=156)(22.373)[MAOB](http://www.ncbi.nlm.nih.gov/gene/?term=4129)(22.373) [SORCS3](http://www.ncbi.nlm.nih.gov/gene/?term=22986)(22.373)[ADRB1](http://www.ncbi.nlm.nih.gov/gene/?term=153)(22.373)[ADAM8](http://www.ncbi.nlm.nih.gov/gene/?term=101)(22.373)[VTI1A](http://www.ncbi.nlm.nih.gov/gene/?term=143187)(22.373)[SLC44A4](http://www.ncbi.nlm.nih.gov/gene/?term=80736)(22.373) [ADAP2](http://www.ncbi.nlm.nih.gov/gene/?term=55803)(22.373)[LRRC8A](http://www.ncbi.nlm.nih.gov/gene/?term=56262)(22.373)[FGFR2](http://www.ncbi.nlm.nih.gov/gene/?term=2263)(22.373)[AURKA](http://www.ncbi.nlm.nih.gov/gene/?term=6790)(22.373)[MCM3](http://www.ncbi.nlm.nih.gov/gene/?term=4172)(22.373) [VCP](http://www.ncbi.nlm.nih.gov/gene/?term=7415)(22.373)[GHRL](http://www.ncbi.nlm.nih.gov/gene/?term=51738)(22.373)[BCL11B](http://www.ncbi.nlm.nih.gov/gene/?term=64919)(22.373)[BCL2](http://www.ncbi.nlm.nih.gov/gene/?term=596)(22.373)[8-Mar](http://www.ncbi.nlm.nih.gov/gene/?term=220972)(22.373) [GAS6](http://www.ncbi.nlm.nih.gov/gene/?term=2621)(22.373)[C5](http://www.ncbi.nlm.nih.gov/gene/?term=727)(22.373)[SREBF1](http://www.ncbi.nlm.nih.gov/gene/?term=6720)(22.373)[DRD2](http://www.ncbi.nlm.nih.gov/gene/?term=1813)(22.373)[PLN](http://www.ncbi.nlm.nih.gov/gene/?term=5350)(22.373) [DNM3](http://www.ncbi.nlm.nih.gov/gene/?term=26052)(22.373)[CHRNA3](http://www.ncbi.nlm.nih.gov/gene/?term=1136)(22.373)[TCF3](http://www.ncbi.nlm.nih.gov/gene/?term=6929)(22.373)[SRC](http://www.ncbi.nlm.nih.gov/gene/?term=6714)(22.373)[LONP1](http://www.ncbi.nlm.nih.gov/gene/?term=9361)(22.373) [XCL1](http://www.ncbi.nlm.nih.gov/gene/?term=6375)(22.373)[HSP90AB1](http://www.ncbi.nlm.nih.gov/gene/?term=3326)(22.373)[HIBADH](http://www.ncbi.nlm.nih.gov/gene/?term=11112)(22.373)[STAP1](http://www.ncbi.nlm.nih.gov/gene/?term=26228)(22.373)[HRC](http://www.ncbi.nlm.nih.gov/gene/?term=3270)(22.373) [UTS2R](http://www.ncbi.nlm.nih.gov/gene/?term=2837)(22.373)[SLC6A4](http://www.ncbi.nlm.nih.gov/gene/?term=6532)(22.373)[HMGCR](http://www.ncbi.nlm.nih.gov/gene/?term=3156)(22.373)[HIPK2](http://www.ncbi.nlm.nih.gov/gene/?term=28996)(22.373)[PPP3CA](http://www.ncbi.nlm.nih.gov/gene/?term=5530)(22.373) [RAPGEF2](http://www.ncbi.nlm.nih.gov/gene/?term=9693)(22.373)[TET1](http://www.ncbi.nlm.nih.gov/gene/?term=80312)(22.373)[C2CD5](http://www.ncbi.nlm.nih.gov/gene/?term=9847)(22.373)[CSF2](http://www.ncbi.nlm.nih.gov/gene/?term=1437)(22.373)[TALDO1](http://www.ncbi.nlm.nih.gov/gene/?term=6888)(22.373) [BDKRB2](http://www.ncbi.nlm.nih.gov/gene/?term=624)(22.373)[KCNH2](http://www.ncbi.nlm.nih.gov/gene/?term=3757)(22.373)[MMP28](http://www.ncbi.nlm.nih.gov/gene/?term=79148)(22.373)[HIF1A](http://www.ncbi.nlm.nih.gov/gene/?term=3091)(22.373)[KIT](http://www.ncbi.nlm.nih.gov/gene/?term=3815)(22.373) [INS](http://www.ncbi.nlm.nih.gov/gene/?term=3630)(22.373)[MTOR](http://www.ncbi.nlm.nih.gov/gene/?term=2475)(22.373)[WNT10B](http://www.ncbi.nlm.nih.gov/gene/?term=7480)(22.373)[SLC18A3](http://www.ncbi.nlm.nih.gov/gene/?term=6572)(22.373)[ABCC4](http://www.ncbi.nlm.nih.gov/gene/?term=10257)(22.373) [BNIP3](http://www.ncbi.nlm.nih.gov/gene/?term=664)(22.373)[ZFPM1](http://www.ncbi.nlm.nih.gov/gene/?term=161882)(22.373)[GATA3](http://www.ncbi.nlm.nih.gov/gene/?term=2625)(22.373)[UBR5](http://www.ncbi.nlm.nih.gov/gene/?term=51366)(22.373)[AREG](http://www.ncbi.nlm.nih.gov/gene/?term=374)(22.373) [CRH](http://www.ncbi.nlm.nih.gov/gene/?term=1392)(22.373)[ZFP42](http://www.ncbi.nlm.nih.gov/gene/?term=132625)(22.373)[PIK3R6](http://www.ncbi.nlm.nih.gov/gene/?term=146850)(22.373)[PF4](http://www.ncbi.nlm.nih.gov/gene/?term=5196)(22.373)[ATP1A2](http://www.ncbi.nlm.nih.gov/gene/?term=477)(22.373) [AVPR2](http://www.ncbi.nlm.nih.gov/gene/?term=554)(22.373)[CAMK2D](http://www.ncbi.nlm.nih.gov/gene/?term=817)(22.373)[ADRA1B](http://www.ncbi.nlm.nih.gov/gene/?term=147)(22.373)[IL10](http://www.ncbi.nlm.nih.gov/gene/?term=3586)(22.373)[NEFL](http://www.ncbi.nlm.nih.gov/gene/?term=4747)(22.373) [UCN2](http://www.ncbi.nlm.nih.gov/gene/?term=90226)(22.373)[ATP1A1](http://www.ncbi.nlm.nih.gov/gene/?term=476)(22.373)[RYR3](http://www.ncbi.nlm.nih.gov/gene/?term=6263)(22.373)[PPP3R1](http://www.ncbi.nlm.nih.gov/gene/?term=5534)(22.373)[SPX](http://www.ncbi.nlm.nih.gov/gene/?term=80763)(22.373) [ATP2A1](http://www.ncbi.nlm.nih.gov/gene/?term=487)(22.373)[SMO](http://www.ncbi.nlm.nih.gov/gene/?term=6608)(22.373)[RAG1](http://www.ncbi.nlm.nih.gov/gene/?term=5896)(22.373)[RAB8B](http://www.ncbi.nlm.nih.gov/gene/?term=51762)(22.373)[DKK3](http://www.ncbi.nlm.nih.gov/gene/?term=27122)(22.373) [AHCYL1](http://www.ncbi.nlm.nih.gov/gene/?term=10768)(22.373)[AQP1](http://www.ncbi.nlm.nih.gov/gene/?term=358)(22.373)[TNFAIP3](http://www.ncbi.nlm.nih.gov/gene/?term=7128)(22.373)[TAC4](http://www.ncbi.nlm.nih.gov/gene/?term=255061)(22.373)[DPPA3](http://www.ncbi.nlm.nih.gov/gene/?term=359787)(22.373) [CASQ1](http://www.ncbi.nlm.nih.gov/gene/?term=844)(22.373)[FKBP1B](http://www.ncbi.nlm.nih.gov/gene/?term=2281)(22.373)[CDC42](http://www.ncbi.nlm.nih.gov/gene/?term=998)(22.373)[IL4](http://www.ncbi.nlm.nih.gov/gene/?term=3565)(22.373)[POLB](http://www.ncbi.nlm.nih.gov/gene/?term=5423)(22.373) [IDO1](http://www.ncbi.nlm.nih.gov/gene/?term=3620)(22.373)[ATP2B4](http://www.ncbi.nlm.nih.gov/gene/?term=493)(22.373)[CTNNB1](http://www.ncbi.nlm.nih.gov/gene/?term=1499)(22.373)[PGLS](http://www.ncbi.nlm.nih.gov/gene/?term=25796)(22.373)[SLC9A3R1](http://www.ncbi.nlm.nih.gov/gene/?term=9368)(22.373) [IGF2](http://www.ncbi.nlm.nih.gov/gene/?term=3481)(22.373)[HPS4](http://www.ncbi.nlm.nih.gov/gene/?term=89781)(22.373)[TKT](http://www.ncbi.nlm.nih.gov/gene/?term=7086)(22.373)[DRD1](http://www.ncbi.nlm.nih.gov/gene/?term=1812)(22.373)[ADAP1](http://www.ncbi.nlm.nih.gov/gene/?term=11033)(22.373) [EDA](http://www.ncbi.nlm.nih.gov/gene/?term=1896)(22.373)[RYR2](http://www.ncbi.nlm.nih.gov/gene/?term=6262)(22.373)[CD34](http://www.ncbi.nlm.nih.gov/gene/?term=947)(22.373)[HSP90AA1](http://www.ncbi.nlm.nih.gov/gene/?term=3320)(22.373)[LTA](http://www.ncbi.nlm.nih.gov/gene/?term=4049)(22.373) [SPR](http://www.ncbi.nlm.nih.gov/gene/?term=6697)(22.373)[GPER1](http://www.ncbi.nlm.nih.gov/gene/?term=2852)(22.373)[CBFA2T3](http://www.ncbi.nlm.nih.gov/gene/?term=863)(22.373)[CALHM1](http://www.ncbi.nlm.nih.gov/gene/?term=255022)(22.373)[WNT5A](http://www.ncbi.nlm.nih.gov/gene/?term=7474)(22.373) [SELP](http://www.ncbi.nlm.nih.gov/gene/?term=6403)(22.373)[XRCC6BP1](http://www.ncbi.nlm.nih.gov/gene/?term=91419)(22.373)[PARP10](http://www.ncbi.nlm.nih.gov/gene/?term=84875)(22.373)[HPRT1](http://www.ncbi.nlm.nih.gov/gene/?term=3251)(22.373)[AMICA1](http://www.ncbi.nlm.nih.gov/gene/?term=120425)(22.373) [TAS1R3](http://www.ncbi.nlm.nih.gov/gene/?term=83756)(22.373)[NOS1](http://www.ncbi.nlm.nih.gov/gene/?term=4842)(22.373)[TREM1](http://www.ncbi.nlm.nih.gov/gene/?term=54210)(22.373)[TAL1](http://www.ncbi.nlm.nih.gov/gene/?term=6886)(22.373)[ALAD](http://www.ncbi.nlm.nih.gov/gene/?term=210)(22.373) [PAWR](http://www.ncbi.nlm.nih.gov/gene/?term=5074)(22.373)[SOX9](http://www.ncbi.nlm.nih.gov/gene/?term=6662)(22.373)[BMP5](http://www.ncbi.nlm.nih.gov/gene/?term=653)(22.373)[PDGFB](http://www.ncbi.nlm.nih.gov/gene/?term=5155)(22.373)[RTN2](http://www.ncbi.nlm.nih.gov/gene/?term=6253)(22.373) [CD63](http://www.ncbi.nlm.nih.gov/gene/?term=967)(22.373)[CAMK2G](http://www.ncbi.nlm.nih.gov/gene/?term=818)(22.373)[LAMP2](http://www.ncbi.nlm.nih.gov/gene/?term=3920)(22.373)[NEFH](http://www.ncbi.nlm.nih.gov/gene/?term=4744)(22.373)[GLYR1](http://www.ncbi.nlm.nih.gov/gene/?term=84656)(22.373) [VDR](http://www.ncbi.nlm.nih.gov/gene/?term=7421)(22.373)[GNAS](http://www.ncbi.nlm.nih.gov/gene/?term=2778)(22.373)[NCBP2](http://www.ncbi.nlm.nih.gov/gene/?term=22916)(22.373)[TPM1](http://www.ncbi.nlm.nih.gov/gene/?term=7168)(22.373)[PIK3R5](http://www.ncbi.nlm.nih.gov/gene/?term=23533)(22.373) [PCSK6](http://www.ncbi.nlm.nih.gov/gene/?term=5046)(22.373)[TRIM24](http://www.ncbi.nlm.nih.gov/gene/?term=8805)(22.373)[REST](http://www.ncbi.nlm.nih.gov/gene/?term=5978)(22.373)[CRHR2](http://www.ncbi.nlm.nih.gov/gene/?term=1395)(22.373)[HCN2](http://www.ncbi.nlm.nih.gov/gene/?term=610)(22.373) [RAG2](http://www.ncbi.nlm.nih.gov/gene/?term=5897)(22.373)[NFX1](http://www.ncbi.nlm.nih.gov/gene/?term=4799)(22.373)[TAS1R2](http://www.ncbi.nlm.nih.gov/gene/?term=80834)(22.373) |
| curdione | [PDE7B](http://www.ncbi.nlm.nih.gov/gene/?term=27115)(80.882)[PDE5A](http://www.ncbi.nlm.nih.gov/gene/?term=8654)(80.882)[PDE9A](http://www.ncbi.nlm.nih.gov/gene/?term=5152)(80.882)[PDE4A](http://www.ncbi.nlm.nih.gov/gene/?term=5141)(80.882)[RYR1](http://www.ncbi.nlm.nih.gov/gene/?term=6261)(80.882) [CYP17A1](http://www.ncbi.nlm.nih.gov/gene/?term=1586)(80.882)[PDE3A](http://www.ncbi.nlm.nih.gov/gene/?term=5139)(80.882)[ADORA2A](http://www.ncbi.nlm.nih.gov/gene/?term=135)(80.882)[ITPR1](http://www.ncbi.nlm.nih.gov/gene/?term=3708)(80.882)[PDE6A](http://www.ncbi.nlm.nih.gov/gene/?term=5145)(80.882) [PRKDC](http://www.ncbi.nlm.nih.gov/gene/?term=5591)(80.882)[PIK3CD](http://www.ncbi.nlm.nih.gov/gene/?term=5293)(80.882)[ESR1](http://www.ncbi.nlm.nih.gov/gene/?term=2099)(80.882)[PDE3B](http://www.ncbi.nlm.nih.gov/gene/?term=5140)(80.882)[PGD](http://www.ncbi.nlm.nih.gov/gene/?term=5226)(80.882) [PIK3CA](http://www.ncbi.nlm.nih.gov/gene/?term=5290)(80.882)[ADORA2B](http://www.ncbi.nlm.nih.gov/gene/?term=136)(80.882)[PDE1A](http://www.ncbi.nlm.nih.gov/gene/?term=5136)(80.882)[PDE4D](http://www.ncbi.nlm.nih.gov/gene/?term=5144)(80.882)[PGR](http://www.ncbi.nlm.nih.gov/gene/?term=5241)(80.882) [PIK3CB](http://www.ncbi.nlm.nih.gov/gene/?term=5291)(80.882)[ITPR2](http://www.ncbi.nlm.nih.gov/gene/?term=3709)(80.882)[PDE1B](http://www.ncbi.nlm.nih.gov/gene/?term=5153)(80.882)[OPRK1](http://www.ncbi.nlm.nih.gov/gene/?term=4986)(80.882)[PDE7A](http://www.ncbi.nlm.nih.gov/gene/?term=5150)(80.882) [ITPR3](http://www.ncbi.nlm.nih.gov/gene/?term=3710)(80.882)[POLA2](http://www.ncbi.nlm.nih.gov/gene/?term=23649)(80.882)[PDE4C](http://www.ncbi.nlm.nih.gov/gene/?term=5143)(80.882)[PDE10A](http://www.ncbi.nlm.nih.gov/gene/?term=10846)(80.882)[PDE1C](http://www.ncbi.nlm.nih.gov/gene/?term=5137)(80.882) [ATM](http://www.ncbi.nlm.nih.gov/gene/?term=472)(80.882)[PDE6B](http://www.ncbi.nlm.nih.gov/gene/?term=5158)(80.882)[PDE4B](http://www.ncbi.nlm.nih.gov/gene/?term=5142)(80.882)[PDE2A](http://www.ncbi.nlm.nih.gov/gene/?term=5138)(80.882)[ADORA1](http://www.ncbi.nlm.nih.gov/gene/?term=134)(80.882) [CYP19A1](http://www.ncbi.nlm.nih.gov/gene/?term=1588)(80.882)[PDE8B](http://www.ncbi.nlm.nih.gov/gene/?term=8622)(80.882)[NT5E](http://www.ncbi.nlm.nih.gov/gene/?term=4907)(80.882)[HDAC2](http://www.ncbi.nlm.nih.gov/gene/?term=3066)(80.882)[PDE6C](http://www.ncbi.nlm.nih.gov/gene/?term=5146)(80.882) [PDE11A](http://www.ncbi.nlm.nih.gov/gene/?term=50940)(80.882)[PDE8A](http://www.ncbi.nlm.nih.gov/gene/?term=5151)(80.882)[NR3C2](http://www.ncbi.nlm.nih.gov/gene/?term=4306)(80.882)[RINT1](http://www.ncbi.nlm.nih.gov/gene/?term=60561)(55.444)[RIPK1](http://www.ncbi.nlm.nih.gov/gene/?term=8737)(55.444) [PIK3R1](http://www.ncbi.nlm.nih.gov/gene/?term=5295)(55.444)[WNT4](http://www.ncbi.nlm.nih.gov/gene/?term=54361)(55.444)[TACR2](http://www.ncbi.nlm.nih.gov/gene/?term=6865)(55.444)[AMPD3](http://www.ncbi.nlm.nih.gov/gene/?term=272)(55.444)[CX3CR1](http://www.ncbi.nlm.nih.gov/gene/?term=1524)(55.444) [ADA](http://www.ncbi.nlm.nih.gov/gene/?term=100)(55.444)[HAP1](http://www.ncbi.nlm.nih.gov/gene/?term=9001)(55.444)[IDNK](http://www.ncbi.nlm.nih.gov/gene/?term=414328)(55.444)[F12](http://www.ncbi.nlm.nih.gov/gene/?term=2161)(48.000)[HDAC1](http://www.ncbi.nlm.nih.gov/gene/?term=3065)(23.000) [TFAP2C](http://www.ncbi.nlm.nih.gov/gene/?term=7022)(22.373)[HMGA2](http://www.ncbi.nlm.nih.gov/gene/?term=8091)(22.373)[TNFSF11](http://www.ncbi.nlm.nih.gov/gene/?term=8600)(22.373)[NT5C1A](http://www.ncbi.nlm.nih.gov/gene/?term=84618)(22.373)[ACTN3](http://www.ncbi.nlm.nih.gov/gene/?term=89)(22.373) [NOS1AP](http://www.ncbi.nlm.nih.gov/gene/?term=9722)(22.373)[TAC1](http://www.ncbi.nlm.nih.gov/gene/?term=6863)(22.373)[PHB](http://www.ncbi.nlm.nih.gov/gene/?term=5245)(22.373)[SIRT1](http://www.ncbi.nlm.nih.gov/gene/?term=23411)(22.373)[DGKI](http://www.ncbi.nlm.nih.gov/gene/?term=9162)(22.373) [FKBP1A](http://www.ncbi.nlm.nih.gov/gene/?term=2280)(22.373)[FURIN](http://www.ncbi.nlm.nih.gov/gene/?term=5045)(22.373)[TOX3](http://www.ncbi.nlm.nih.gov/gene/?term=27324)(22.373)[UTS2](http://www.ncbi.nlm.nih.gov/gene/?term=10911)(22.373)[STUB1](http://www.ncbi.nlm.nih.gov/gene/?term=10273)(22.373) [CTR9](http://www.ncbi.nlm.nih.gov/gene/?term=9646)(22.373)[G6PD](http://www.ncbi.nlm.nih.gov/gene/?term=2539)(22.373)[GNAT1](http://www.ncbi.nlm.nih.gov/gene/?term=2779)(22.373)[SIX1](http://www.ncbi.nlm.nih.gov/gene/?term=6495)(22.373)[QDPR](http://www.ncbi.nlm.nih.gov/gene/?term=5860)(22.373) [TGFB1](http://www.ncbi.nlm.nih.gov/gene/?term=7040)(22.373)[ATP11C](http://www.ncbi.nlm.nih.gov/gene/?term=286410)(22.373)[KHDRBS1](http://www.ncbi.nlm.nih.gov/gene/?term=10657)(22.373)[PPP3CB](http://www.ncbi.nlm.nih.gov/gene/?term=5532)(22.373)[SYT2](http://www.ncbi.nlm.nih.gov/gene/?term=127833)(22.373) [SIX4](http://www.ncbi.nlm.nih.gov/gene/?term=51804)(22.373)[SIRT2](http://www.ncbi.nlm.nih.gov/gene/?term=22933)(22.373)[SPI1](http://www.ncbi.nlm.nih.gov/gene/?term=6688)(22.373)[HOMER1](http://www.ncbi.nlm.nih.gov/gene/?term=9456)(22.373)[SHANK3](http://www.ncbi.nlm.nih.gov/gene/?term=85358)(22.373) [TAS1R1](http://www.ncbi.nlm.nih.gov/gene/?term=80835)(22.373)[TRIM28](http://www.ncbi.nlm.nih.gov/gene/?term=10155)(22.373)[XRCC4](http://www.ncbi.nlm.nih.gov/gene/?term=7518)(22.373)[MED1](http://www.ncbi.nlm.nih.gov/gene/?term=5469)(22.373)[NKX3-1](http://www.ncbi.nlm.nih.gov/gene/?term=4824)(22.373) [UBE2B](http://www.ncbi.nlm.nih.gov/gene/?term=7320)(22.373)[ESR2](http://www.ncbi.nlm.nih.gov/gene/?term=2100)(22.373)[HCN4](http://www.ncbi.nlm.nih.gov/gene/?term=10021)(22.373)[EDN1](http://www.ncbi.nlm.nih.gov/gene/?term=1906)(22.373)[CACNA1A](http://www.ncbi.nlm.nih.gov/gene/?term=773)(22.373) [STAT5A](http://www.ncbi.nlm.nih.gov/gene/?term=6776)(22.373)[TP53](http://www.ncbi.nlm.nih.gov/gene/?term=7157)(22.373)[GNAT3](http://www.ncbi.nlm.nih.gov/gene/?term=346562)(22.373)[HELB](http://www.ncbi.nlm.nih.gov/gene/?term=92797)(22.373)[COMT](http://www.ncbi.nlm.nih.gov/gene/?term=1312)(22.373) [SLC26A6](http://www.ncbi.nlm.nih.gov/gene/?term=65010)(22.373)[ADRA2A](http://www.ncbi.nlm.nih.gov/gene/?term=150)(22.373)[YWHAE](http://www.ncbi.nlm.nih.gov/gene/?term=7531)(22.373)[CETN2](http://www.ncbi.nlm.nih.gov/gene/?term=1069)(22.373)[POLA1](http://www.ncbi.nlm.nih.gov/gene/?term=5422)(22.373) [CRP](http://www.ncbi.nlm.nih.gov/gene/?term=1401)(22.373)[CETN1](http://www.ncbi.nlm.nih.gov/gene/?term=1068)(22.373)[NUDT9](http://www.ncbi.nlm.nih.gov/gene/?term=53343)(22.373)[PIK3CG](http://www.ncbi.nlm.nih.gov/gene/?term=5294)(22.373)[BMP2](http://www.ncbi.nlm.nih.gov/gene/?term=650)(22.373) [TNF](http://www.ncbi.nlm.nih.gov/gene/?term=7124)(22.373)[NEDD4](http://www.ncbi.nlm.nih.gov/gene/?term=4734)(22.373)[AGT](http://www.ncbi.nlm.nih.gov/gene/?term=183)(22.373)[FER](http://www.ncbi.nlm.nih.gov/gene/?term=2241)(22.373)[SHH](http://www.ncbi.nlm.nih.gov/gene/?term=6469)(22.373) [SERPINB3](http://www.ncbi.nlm.nih.gov/gene/?term=6317)(22.373)[FOXP3](http://www.ncbi.nlm.nih.gov/gene/?term=50943)(22.373)[CXCL13](http://www.ncbi.nlm.nih.gov/gene/?term=10563)(22.373)[ARID1A](http://www.ncbi.nlm.nih.gov/gene/?term=8289)(22.373)[FGF10](http://www.ncbi.nlm.nih.gov/gene/?term=2255)(22.373) [KCNB1](http://www.ncbi.nlm.nih.gov/gene/?term=3745)(22.373)[LEF1](http://www.ncbi.nlm.nih.gov/gene/?term=51176)(22.373)[PTK2B](http://www.ncbi.nlm.nih.gov/gene/?term=2185)(22.373)[LIG4](http://www.ncbi.nlm.nih.gov/gene/?term=3981)(22.373)[ADRBK1](http://www.ncbi.nlm.nih.gov/gene/?term=156)(22.373) [MAOB](http://www.ncbi.nlm.nih.gov/gene/?term=4129)(22.373)[SORCS3](http://www.ncbi.nlm.nih.gov/gene/?term=22986)(22.373)[ADRB1](http://www.ncbi.nlm.nih.gov/gene/?term=153)(22.373)[ADAM8](http://www.ncbi.nlm.nih.gov/gene/?term=101)(22.373)[VTI1A](http://www.ncbi.nlm.nih.gov/gene/?term=143187)(22.373) [SLC44A4](http://www.ncbi.nlm.nih.gov/gene/?term=80736)(22.373)[ADAP2](http://www.ncbi.nlm.nih.gov/gene/?term=55803)(22.373)[LRRC8A](http://www.ncbi.nlm.nih.gov/gene/?term=56262)(22.373)[FGFR2](http://www.ncbi.nlm.nih.gov/gene/?term=2263)(22.373)[AURKA](http://www.ncbi.nlm.nih.gov/gene/?term=6790)(22.373) [MCM3](http://www.ncbi.nlm.nih.gov/gene/?term=4172)(22.373)[VCP](http://www.ncbi.nlm.nih.gov/gene/?term=7415)(22.373)[GHRL](http://www.ncbi.nlm.nih.gov/gene/?term=51738)(22.373)[BCL11B](http://www.ncbi.nlm.nih.gov/gene/?term=64919)(22.373)[BCL2](http://www.ncbi.nlm.nih.gov/gene/?term=596)(22.373) [8-Mar](http://www.ncbi.nlm.nih.gov/gene/?term=220972)(22.373)[GAS6](http://www.ncbi.nlm.nih.gov/gene/?term=2621)(22.373)[C5](http://www.ncbi.nlm.nih.gov/gene/?term=727)(22.373)[SREBF1](http://www.ncbi.nlm.nih.gov/gene/?term=6720)(22.373)[DRD2](http://www.ncbi.nlm.nih.gov/gene/?term=1813)(22.373) [PLN](http://www.ncbi.nlm.nih.gov/gene/?term=5350)(22.373)[DNM3](http://www.ncbi.nlm.nih.gov/gene/?term=26052)(22.373)[CHRNA3](http://www.ncbi.nlm.nih.gov/gene/?term=1136)(22.373)[TCF3](http://www.ncbi.nlm.nih.gov/gene/?term=6929)(22.373)[SRC](http://www.ncbi.nlm.nih.gov/gene/?term=6714)(22.373) [LONP1](http://www.ncbi.nlm.nih.gov/gene/?term=9361)(22.373)[XCL1](http://www.ncbi.nlm.nih.gov/gene/?term=6375)(22.373)[HSP90AB1](http://www.ncbi.nlm.nih.gov/gene/?term=3326)(22.373)[HIBADH](http://www.ncbi.nlm.nih.gov/gene/?term=11112)(22.373)[STAP1](http://www.ncbi.nlm.nih.gov/gene/?term=26228)(22.373) [HRC](http://www.ncbi.nlm.nih.gov/gene/?term=3270)(22.373)[UTS2R](http://www.ncbi.nlm.nih.gov/gene/?term=2837)(22.373)[SLC6A4](http://www.ncbi.nlm.nih.gov/gene/?term=6532)(22.373)[HMGCR](http://www.ncbi.nlm.nih.gov/gene/?term=3156)(22.373)[HIPK2](http://www.ncbi.nlm.nih.gov/gene/?term=28996)(22.373) [PPP3CA](http://www.ncbi.nlm.nih.gov/gene/?term=5530)(22.373)[RAPGEF2](http://www.ncbi.nlm.nih.gov/gene/?term=9693)(22.373)[TET1](http://www.ncbi.nlm.nih.gov/gene/?term=80312)(22.373)[C2CD5](http://www.ncbi.nlm.nih.gov/gene/?term=9847)(22.373)[CSF2](http://www.ncbi.nlm.nih.gov/gene/?term=1437)(22.373) [TALDO1](http://www.ncbi.nlm.nih.gov/gene/?term=6888)(22.373)[BDKRB2](http://www.ncbi.nlm.nih.gov/gene/?term=624)(22.373)[KCNH2](http://www.ncbi.nlm.nih.gov/gene/?term=3757)(22.373)[MMP28](http://www.ncbi.nlm.nih.gov/gene/?term=79148)(22.373)[HIF1A](http://www.ncbi.nlm.nih.gov/gene/?term=3091)(22.373) [KIT](http://www.ncbi.nlm.nih.gov/gene/?term=3815)(22.373)[INS](http://www.ncbi.nlm.nih.gov/gene/?term=3630)(22.373)[MTOR](http://www.ncbi.nlm.nih.gov/gene/?term=2475)(22.373)[WNT10B](http://www.ncbi.nlm.nih.gov/gene/?term=7480)(22.373)[SLC18A3](http://www.ncbi.nlm.nih.gov/gene/?term=6572)(22.373) [ABCC4](http://www.ncbi.nlm.nih.gov/gene/?term=10257)(22.373)[BNIP3](http://www.ncbi.nlm.nih.gov/gene/?term=664)(22.373)[ZFPM1](http://www.ncbi.nlm.nih.gov/gene/?term=161882)(22.373)[GATA3](http://www.ncbi.nlm.nih.gov/gene/?term=2625)(22.373)[UBR5](http://www.ncbi.nlm.nih.gov/gene/?term=51366)(22.373) [AREG](http://www.ncbi.nlm.nih.gov/gene/?term=374)(22.373)[CRH](http://www.ncbi.nlm.nih.gov/gene/?term=1392)(22.373)[ZFP42](http://www.ncbi.nlm.nih.gov/gene/?term=132625)(22.373)[PIK3R6](http://www.ncbi.nlm.nih.gov/gene/?term=146850)(22.373)[PF4](http://www.ncbi.nlm.nih.gov/gene/?term=5196)(22.373) [ATP1A2](http://www.ncbi.nlm.nih.gov/gene/?term=477)(22.373)[AVPR2](http://www.ncbi.nlm.nih.gov/gene/?term=554)(22.373)[CAMK2D](http://www.ncbi.nlm.nih.gov/gene/?term=817)(22.373)[ADRA1B](http://www.ncbi.nlm.nih.gov/gene/?term=147)(22.373)[IL10](http://www.ncbi.nlm.nih.gov/gene/?term=3586)(22.373) [NEFL](http://www.ncbi.nlm.nih.gov/gene/?term=4747)(22.373)[UCN2](http://www.ncbi.nlm.nih.gov/gene/?term=90226)(22.373)[ATP1A1](http://www.ncbi.nlm.nih.gov/gene/?term=476)(22.373)[RYR3](http://www.ncbi.nlm.nih.gov/gene/?term=6263)(22.373)[PPP3R1](http://www.ncbi.nlm.nih.gov/gene/?term=5534)(22.373) [SPX](http://www.ncbi.nlm.nih.gov/gene/?term=80763)(22.373)[ATP2A1](http://www.ncbi.nlm.nih.gov/gene/?term=487)(22.373)[SMO](http://www.ncbi.nlm.nih.gov/gene/?term=6608)(22.373)[RAG1](http://www.ncbi.nlm.nih.gov/gene/?term=5896)(22.373)[RAB8B](http://www.ncbi.nlm.nih.gov/gene/?term=51762)(22.373) [DKK3](http://www.ncbi.nlm.nih.gov/gene/?term=27122)(22.373)[AHCYL1](http://www.ncbi.nlm.nih.gov/gene/?term=10768)(22.373)[AQP1](http://www.ncbi.nlm.nih.gov/gene/?term=358)(22.373)[TNFAIP3](http://www.ncbi.nlm.nih.gov/gene/?term=7128)(22.373)[TAC4](http://www.ncbi.nlm.nih.gov/gene/?term=255061)(22.373) [DPPA3](http://www.ncbi.nlm.nih.gov/gene/?term=359787)(22.373)[CASQ1](http://www.ncbi.nlm.nih.gov/gene/?term=844)(22.373)[FKBP1B](http://www.ncbi.nlm.nih.gov/gene/?term=2281)(22.373)[CDC42](http://www.ncbi.nlm.nih.gov/gene/?term=998)(22.373)[IL4](http://www.ncbi.nlm.nih.gov/gene/?term=3565)(22.373) [POLB](http://www.ncbi.nlm.nih.gov/gene/?term=5423)(22.373)[IDO1](http://www.ncbi.nlm.nih.gov/gene/?term=3620)(22.373)[ATP2B4](http://www.ncbi.nlm.nih.gov/gene/?term=493)(22.373)[CTNNB1](http://www.ncbi.nlm.nih.gov/gene/?term=1499)(22.373)[PGLS](http://www.ncbi.nlm.nih.gov/gene/?term=25796)(22.373) [SLC9A3R1](http://www.ncbi.nlm.nih.gov/gene/?term=9368)(22.373)[IGF2](http://www.ncbi.nlm.nih.gov/gene/?term=3481)(22.373)[HPS4](http://www.ncbi.nlm.nih.gov/gene/?term=89781)(22.373)[TKT](http://www.ncbi.nlm.nih.gov/gene/?term=7086)(22.373)[DRD1](http://www.ncbi.nlm.nih.gov/gene/?term=1812)(22.373) [ADAP1](http://www.ncbi.nlm.nih.gov/gene/?term=11033)(22.373)[EDA](http://www.ncbi.nlm.nih.gov/gene/?term=1896)(22.373)[RYR2](http://www.ncbi.nlm.nih.gov/gene/?term=6262)(22.373)[CD34](http://www.ncbi.nlm.nih.gov/gene/?term=947)(22.373)[HSP90AA1](http://www.ncbi.nlm.nih.gov/gene/?term=3320)(22.373) [LTA](http://www.ncbi.nlm.nih.gov/gene/?term=4049)(22.373)[SPR](http://www.ncbi.nlm.nih.gov/gene/?term=6697)(22.373)[GPER1](http://www.ncbi.nlm.nih.gov/gene/?term=2852)(22.373)[CBFA2T3](http://www.ncbi.nlm.nih.gov/gene/?term=863)(22.373)[CALHM1](http://www.ncbi.nlm.nih.gov/gene/?term=255022)(22.373) [WNT5A](http://www.ncbi.nlm.nih.gov/gene/?term=7474)(22.373)[SELP](http://www.ncbi.nlm.nih.gov/gene/?term=6403)(22.373)[XRCC6BP1](http://www.ncbi.nlm.nih.gov/gene/?term=91419)(22.373)[PARP10](http://www.ncbi.nlm.nih.gov/gene/?term=84875)(22.373)[HPRT1](http://www.ncbi.nlm.nih.gov/gene/?term=3251)(22.373) [AMICA1](http://www.ncbi.nlm.nih.gov/gene/?term=120425)(22.373)[TAS1R3](http://www.ncbi.nlm.nih.gov/gene/?term=83756)(22.373)[NOS1](http://www.ncbi.nlm.nih.gov/gene/?term=4842)(22.373)[TREM1](http://www.ncbi.nlm.nih.gov/gene/?term=54210)(22.373)[TAL1](http://www.ncbi.nlm.nih.gov/gene/?term=6886)(22.373) [ALAD](http://www.ncbi.nlm.nih.gov/gene/?term=210)(22.373)[PAWR](http://www.ncbi.nlm.nih.gov/gene/?term=5074)(22.373)[SOX9](http://www.ncbi.nlm.nih.gov/gene/?term=6662)(22.373)[BMP5](http://www.ncbi.nlm.nih.gov/gene/?term=653)(22.373)[PDGFB](http://www.ncbi.nlm.nih.gov/gene/?term=5155)(22.373) [RTN2](http://www.ncbi.nlm.nih.gov/gene/?term=6253)(22.373)[CD63](http://www.ncbi.nlm.nih.gov/gene/?term=967)(22.373)[CAMK2G](http://www.ncbi.nlm.nih.gov/gene/?term=818)(22.373)[LAMP2](http://www.ncbi.nlm.nih.gov/gene/?term=3920)(22.373)[NEFH](http://www.ncbi.nlm.nih.gov/gene/?term=4744)(22.373) [GLYR1](http://www.ncbi.nlm.nih.gov/gene/?term=84656)(22.373)[VDR](http://www.ncbi.nlm.nih.gov/gene/?term=7421)(22.373)[GNAS](http://www.ncbi.nlm.nih.gov/gene/?term=2778)(22.373)[NCBP2](http://www.ncbi.nlm.nih.gov/gene/?term=22916)(22.373)[TPM1](http://www.ncbi.nlm.nih.gov/gene/?term=7168)(22.373) [PIK3R5](http://www.ncbi.nlm.nih.gov/gene/?term=23533)(22.373)[PCSK6](http://www.ncbi.nlm.nih.gov/gene/?term=5046)(22.373)[TRIM24](http://www.ncbi.nlm.nih.gov/gene/?term=8805)(22.373)[REST](http://www.ncbi.nlm.nih.gov/gene/?term=5978)(22.373)[CRHR2](http://www.ncbi.nlm.nih.gov/gene/?term=1395)(22.373) [HCN2](http://www.ncbi.nlm.nih.gov/gene/?term=610)(22.373)[RAG2](http://www.ncbi.nlm.nih.gov/gene/?term=5897)(22.373)[NFX1](http://www.ncbi.nlm.nih.gov/gene/?term=4799)(22.373)[TAS1R2](http://www.ncbi.nlm.nih.gov/gene/?term=80834)(22.373) |
| furanodiene | This compound doesn't have any potential target with score larger than 20. |

Note: As set, for each query TCM’s compositive compound, only the predicted candidate target proteins with scores >= 20 are presented.

**Supplementary table 2. Results from BATMAN-TCM of TTD diseases database for Target Genes/proteins from Germacrone, Curdione.**

| **TTD ID** | **Adjusted p-value** | **Target Genes/proteins**^*^ |
| --- | --- | --- |
| Cancer, unspecific | 9.80e-001 | HDAC1; KIT; MTOR; PDGFB; PIK3CG; SRC; TP53; |
| Breast cancer | 1.08e-001 | CYP19A1; ESR1; ESR2; HSP90AA1; PGR; SRC; VDR; |
| Prostate cancer | 2.24e-001 | CYP17A1; HSP90AA1; TGFB1; TP53; VDR; |
| Osteosarcoma | 1.36e-003 | PDE4A; PDE4B; PDE4C; PDE4D; |
| Solid tumors | 3.61e-001 | AURKA; HSP90AA1; PIK3CG; |
| Ovarian cancer | 2.42e-001 | HSP90AA1; IGF2; KIT; |
| Inflammation | 3.61e-001 | ADAM8; ADORA1; ADORA2A; CSF2; KIT; |
| Asthma | 7.02e-004 | ADORA1; ADORA2B; ADRB1; BDKRB2; IL4; PDE4A; PDE4B; PDE4C; PDE4D; TACR2; TNF; |
| Cardiovascular disease | 1.79e-003 | ADRB1;BDKRB2;EDN1;ESR1;ESR2;HMGCR;PDE1A;PDE1B;PDE1C;UTS2 |
| Heart failure | 7.67e-004 | ADRA2A; ADRBK1; ATP1A1; ATP1A2; PIK3CG; PLN; TNF; |
| Congestive heart failure | 1.33e-004 | ADRBK1; AVPR2; EDN1; TNF; UTS2R; |
| Myocardial infarction | 1.75e-002 | BDKRB2; HMGCR; PIK3CG; UTS2R; |
| Cardiac arrhythmias | 6.13e-002 | ADORA1; ADRB1; KCNH2; |
| Hypertension | 1.58e-002 | ADRA2A; ADRB1; ADRBK1; BDKRB2; ITPR1; ITPR2; ITPR3; |
| Analgesics | 2.99e-001 | ADORA1; ADORA2A; BDKRB2; CACNA1A; OPRK1; TACR2; |
| Erectile dysfunction | 1.33e-004 | DRD2; PDE1A; PDE1B; PDE1C; PDE2A; PDE5A; |
| Neurodegenerative diseases | 6.68e-002 | ADORA2A; CYP19A1; ESR1; ESR2; OPRK1; |
| Parkinson's disease | 1.08e-001 | ADORA2A; COMT; DRD1; DRD2; MAOB; |
| Brain injury | 1.54e-002 | C5; IL4; TNF; |
| Chronic obstructive pulmonary disease | 1.36e-003 | PDE4A; PDE4B; PDE4C; PDE4D; |
| Depression | 1.08e-001 | ADORA2A; DRD2; IDO1; TACR2; |
| Noninsulin-dependent diabetes mellitus | 1.97e-001 | ADORA1; GHRL; KCNB1; TNF; |
| Osteoporosis | 1.08e-001 | ESR1; SRC; TNFSF11; |
| Schizophrenia | 1.08e-001 | DRD2; NOS1; SLC6A4; |
| Rheumatoid arthritis | 4.20e-001 | C5; IL4; TNF; |
| Malignant hyperthermia | 1.79e-003 | RYR1; RYR2; RYR3; |

*Annotation: “Targets” are referred to as the targets mapped to this term, same to table 3.

Supplementary Table 3. Results from BATMAN-TCM of OMIM diseases database for Target Genes/proteins from Germacrone, Curdione.

| **OMIM ID** | **Disease name** | **Adjusted p-value** | **Target Genes/proteins**^*^ |
| --- | --- | --- | --- |
| [OMIM：114480](http://www.omim.org/entry/114480) | Breast Cancer | 2.38e-002 | ATM; ESR1; PHB; PIK3CA; TP53; |
| [OMIM：114500](http://www.omim.org/entry/114500) | Colorectal Cancer | 3.02e-002 | AURKA; CTNNB1; PIK3CA; TP53; |
| [OMIM：114550](http://www.omim.org/entry/114550) | Hepatocellular Carcinoma | 3.02e-002 | CTNNB1; PIK3CA; TP53; |
| [OMIM：167000](http://www.omim.org/entry/167000) | Ovarian Cancer | 3.02e-002 | CTNNB1; PIK3CA; |
| [OMIM：613659](http://www.omim.org/entry/613659) | Gastric Cancer | 3.02e-002 | FGFR2; PIK3CA; |
| [OMIM：611162](http://www.omim.org/entry/611162) | Malaria | 5.79e-002 | G6PD; TNF; |
| [OMIM：609423](http://www.omim.org/entry/609423) | Human Immunodeficiency Virus Type 1 | 5.06e-002 | CX3CR1; IL10; |
| [OMIM：603554](http://www.omim.org/entry/603554) | Omenn Syndrome | 3.02e-002 | RAG1; RAG2; |
| [OMIM：601457](http://www.omim.org/entry/601457) | Severe Combined Immunodeficiency | 3.02e-002 | RAG1; RAG2; |
| [OMIM：608446](http://www.omim.org/entry/608446) | Myocardial Infarction | 4.44e-002 | ESR1; LTA; |
| [OMIM：222100](http://www.omim.org/entry/222100) | Diabetes Mellitus, Insulin-Dependent | 3.02e-002 | FOXP3; ITPR3; |

**Supplementary table 4. Results from BATMAN-TCM of pathways for Target Genes/proteins from Germacrone, Curdione.**

| **KEGG ID** | **KEGG pathways** | **Adjusted p Value** | **Targets*** **Num** | **Targets mapped** |
| --- | --- | --- | --- | --- |
| [hsa00230](http://www.kegg.jp/kegg-bin/show_pathway?hsa00230) | Purine Metabolism | 5.79e-013 | 29 | ADA;AMPD3;HPRT1;NT5C1A;NT5E;NUDT9;PDE10A;PDE11A;PDE1A;PDE1B;PDE1C;PDE2A;PDE3A;PDE3B;PDE4A;PDE4B;PDE4C;PDE4D;PDE5A;PDE6A;PDE6B;PDE6C;PDE7A;PDE7B;PDE8A;PDE8B;PDE9A;POLA1;POLA2; |
| [hsa04020](http://www.kegg.jp/kegg-bin/show_pathway?hsa04020) | Calcium Signaling Pathway | 4.86e-011 | 27 | ADORA2A;ADORA2B;ADRA1B;ADRB1;ATP2A1;ATP2B4;BDKRB2;CACNA1A;CAMK2D;CAMK2G;DRD1;ITPR1;ITPR2;ITPR3;NOS1;PDE1A;PDE1B;PDE1C;PLN;PPP3CA;PPP3CB;PPP3R1;PTK2B;RYR1;RYR2;RYR3;TACR2; |
| [hsa04022](http://www.kegg.jp/kegg-bin/show_pathway?hsa04022) | CGMP - PKG Signaling Pathway | 3.30e-007 | 21 | ADORA1;ADRA1B;ADRA2A;ADRB1;ATP1A1;ATP1A2;ATP2A1;ATP2B4;BDKRB2;INS;ITPR1;ITPR2;ITPR3;PDE2A;PDE3A;PDE3B;PDE5A;PLN;PPP3CA;PPP3CB;PPP3R1; |
| [hsa04080](http://www.kegg.jp/kegg-bin/show_pathway?hsa04080) | Neuroactive Ligand-Receptor Interaction | 5.23e-002 | 15 | ADORA1;ADORA2A;ADORA2B;ADRA1B;ADRA2A;ADRB1;AVPR2;BDKRB2;CHRNA3;CRHR2;DRD1;DRD2;OPRK1;TACR2;UTS2R; |
| [hsa04060](http://www.kegg.jp/kegg-bin/show_pathway?hsa04060) | Cytokine-Cytokine Receptor Interaction | 7.61e-002 | 14 | BMP2;CSF2;CX3CR1;CXCL13;EDA;IL10;IL4;KIT;LTA;PDGFB;PF4;TGFB1;TNFSF11;XCL1; |
| [hsa04015](http://www.kegg.jp/kegg-bin/show_pathway?hsa04015) | Rap1 Signaling Pathway | 7.49e-002 | 12 | ADORA2A;ADORA2B;CDC42;CTNNB1;DRD2;FGF10;FGFR2;INS;KIT;PDGFB;RAPGEF2;SRC; |
| [hsa04114](http://www.kegg.jp/kegg-bin/show_pathway?hsa04114) | Oocyte Meiosis | 1.66e-003 | 12 | AURKA;CAMK2D;CAMK2G;INS;ITPR1;ITPR2;ITPR3;PGR;PPP3CA;PPP3CB;PPP3R1;YWHAE; |
| [hsa04728](http://www.kegg.jp/kegg-bin/show_pathway?hsa04728) | Dopaminergic Synapse | 4.80e-003 | 12 | CACNA1A;CAMK2D;CAMK2G;COMT;DRD1;DRD2;ITPR1;ITPR2;ITPR3;MAOB;PPP3CA;PPP3CB; |
| [hsa04919](http://www.kegg.jp/kegg-bin/show_pathway?hsa04919) | Thyroid Hormone Signaling Pathway | 2.77e-003 | 12 | ATP1A1;ATP1A2;CTNNB1;ESR1;HDAC1;HDAC2;HIF1A;MED1;MTOR;PLN;SRC;WNT4; |
| [hsa04921](http://www.kegg.jp/kegg-bin/show_pathway?hsa04921) | Oxytocin Signaling Pathway | 1.71e-002 | 12 | CAMK2D;CAMK2G;ITPR1;ITPR2;ITPR3;PPP3CA;PPP3CB;PPP3R1;RYR1;RYR2;RYR3;SRC; |
| [hsa04151](http://www.kegg.jp/kegg-bin/show_pathway?hsa04151) | PI3K-Akt Signaling Pathway | 6.70e-001 | 11 | BCL2;FGF10;FGFR2;HSP90AA1;HSP90AB1;IL4;INS;KIT;MTOR;PDGFB;YWHAE; |
| [hsa04261](http://www.kegg.jp/kegg-bin/show_pathway?hsa04261) | Adrenergic Signaling In Cardiomyocytes | 2.38e-002 | 11 | ADRA1B;ADRB1;ATP1A1;ATP1A2;ATP2B4;BCL2;CAMK2D;CAMK2G;PLN;RYR2;TPM1 |
| [hsa04010](http://www.kegg.jp/kegg-bin/show_pathway?hsa04010) | MAPK Signaling Pathway | 4.40e-001 | 10 | CACNA1A;CDC42;FGF10;FGFR2;PDGFB;PPP3CA;PPP3CB;PPP3R1;RAPGEF2;TGFB1; |
| [hsa04310](http://www.kegg.jp/kegg-bin/show_pathway?hsa04310) | Wnt Signaling Pathway | 3.10e-002 | 10 | CAMK2D;CAMK2G;CTNNB1;LEF1;PPP3CA;PPP3CB;PPP3R1;WNT10B;WNT4;WNT5A; |
| [hsa04390](http://www.kegg.jp/kegg-bin/show_pathway?hsa04390) | Hippo Signaling Pathway | 5.23e-002 | 10 | AREG;BMP2;BMP5;CTNNB1;LEF1;TGFB1;WNT10B;WNT4;WNT5A;YWHAE; |
| [hsa04724](http://www.kegg.jp/kegg-bin/show_pathway?hsa04724) | Glutamatergic Synapse | 1.49e-002 | 10 | ADRBK1;CACNA1A;HOMER1;ITPR1;ITPR2;ITPR3;PPP3CA;PPP3CB;PPP3R1;SHANK3; |
| [hsa04970](http://www.kegg.jp/kegg-bin/show_pathway?hsa04970) | Salivary Secretion | 3.59e-003 | 10 | ADRA1B;ADRB1;ATP1A1;ATP1A2;ATP2B4;ITPR1;ITPR2;ITPR3;NOS1;RYR3; |
| [hsa04068](http://www.kegg.jp/kegg-bin/show_pathway?hsa04068) | FoxO Signaling Pathway | 5.41e-002 | 9 | ATM;BNIP3;HOMER1;IL10;INS;RAG1;RAG2;SIRT1;TGFB1; |
| [hsa04144](http://www.kegg.jp/kegg-bin/show_pathway?hsa04144) | Endocytosis | 3.27e-001 | 9 | ADRB1;ADRBK1;CDC42;DNM3;FGFR2;KIT;NEDD4;SRC;TGFB1; |
| [hsa04713](http://www.kegg.jp/kegg-bin/show_pathway?hsa04713) | Circadian Entrainment | 1.49e-002 | 9 | CAMK2D;CAMK2G;ITPR1;ITPR3;NOS1;NOS1AP;RYR1;RYR2;RYR3; |
| [hsa04725](http://www.kegg.jp/kegg-bin/show_pathway?hsa04725) | Cholinergic Synapse | 2.69e-002 | 9 | BCL2;CACNA1A;CAMK2D;CAMK2G;CHRNA3;ITPR1;ITPR2;ITPR3;SLC18A3; |
| [hsa04915](http://www.kegg.jp/kegg-bin/show_pathway?hsa04915) | Estrogen Signaling Pathway | 1.75e-002 | 9 | ESR1;ESR2;GPER1;HSP90AA1;HSP90AB1;ITPR1;ITPR2;ITPR3;SRC |
| [hsa04916](http://www.kegg.jp/kegg-bin/show_pathway?hsa04916) | Melanogenesis | 1.75e-002 | 9 | CAMK2D;CAMK2G;CTNNB1;EDN1;KIT;LEF1;WNT10B;WNT4;WNT5A; |
| [hsa04062](http://www.kegg.jp/kegg-bin/show_pathway?hsa04062) | Chemokine Signaling Pathway | 4.15e-001 | 8 | ADRBK1;CDC42;CX3CR1;CXCL13;PF4;PTK2B;SRC;XCL1; |
| [hsa04540](http://www.kegg.jp/kegg-bin/show_pathway?hsa04540) | Gap Junction | 2.43e-002 | 8 | ADRB1;DRD1;DRD2;ITPR1;ITPR2;ITPR3;PDGFB;SRC; |
| [hsa04720](http://www.kegg.jp/kegg-bin/show_pathway?hsa04720) | Long-Term Potentiation | 7.43e-003 | 8 | CAMK2D;CAMK2G;ITPR1;ITPR2;ITPR3;PPP3CA;PPP3CB;PPP3R1; |
| [hsa04742](http://www.kegg.jp/kegg-bin/show_pathway?hsa04742) | Taste Transduction | 1.89e-003 | 8 | CACNA1A;GNAT3;ITPR3;KCNB1;PDE1A;TAS1R1;TAS1R2;TAS1R3; |
| [hsa04810](http://www.kegg.jp/kegg-bin/show_pathway?hsa04810) | Regulation Of Actin Cytoskeleton | 531e-001 | 8 | ACTN3;BDKRB2;CDC42;FGF10;FGFR2;INS;PDGFB;SRC; |
| [hsa04912](http://www.kegg.jp/kegg-bin/show_pathway?hsa04912) | GnRH Signaling Pathway | 2.69e-002 | 8 | CAMK2D;CAMK2G;CDC42;ITPR1;ITPR2;ITPR3;PTK2B;SRC; |
| [hsa04972](http://www.kegg.jp/kegg-bin/show_pathway?hsa04972) | Pancreatic Secretion | 2.77e-002 | 8 | ATP1A1;ATP1A2;ATP2A1;ATP2B4;ITPR1;ITPR2;ITPR3;RYR2; |
| [hsa04066](http://www.kegg.jp/kegg-bin/show_pathway?hsa04066) | HIF-1 Signaling Pathway | 1.04e-001 | 7 | BCL2;CAMK2D;CAMK2G;EDN1;HIF1A;INS;MTOR; |
| [hsa04110](http://www.kegg.jp/kegg-bin/show_pathway?hsa04110) | Cell Cycle | 1.79e-001 | 7 | ATM;HDAC1;HDAC2;MCM3;PRKDC;TGFB1;YWHAE; |
| [hsa04660](http://www.kegg.jp/kegg-bin/show_pathway?hsa04660) | T Cell Receptor Signaling Pathway | 9.82e-002 | 7 | CDC42;CSF2;IL10;IL4;PPP3CA;PPP3CB;PPP3R1; |
| [hsa04730](http://www.kegg.jp/kegg-bin/show_pathway?hsa04730) | Long-Term Depression | 1.49e-002 | 7 | CACNA1A;CRH;ITPR1;ITPR2;ITPR3;NOS1;RYR1; |
| [hsa04750](http://www.kegg.jp/kegg-bin/show_pathway?hsa04750) | Inflammatory Mediator Regulation Of TRP Channels | 8.44e-002 | 7 | BDKRB2;CAMK2D;CAMK2G;ITPR1;ITPR2;ITPR3;SRC; |
| [hsa04911](http://www.kegg.jp/kegg-bin/show_pathway?hsa04911) | Insulin Secretion | 5.09e-002 | 7 | ATP1A1;ATP1A2;CAMK2D;CAMK2G;INS;ITPR3;RYR2; |
| [hsa04917](http://www.kegg.jp/kegg-bin/show_pathway?hsa04917) | Prolactin Signaling Pathway | 2.65e-002 | 7 | CYP17A1;ESR1;ESR2;INS;SRC;STAT5A;TNFSF11; |
| [Hsa04971](http://www.kegg.jp/kegg-bin/show_pathway?hsa04971) | Gastric Acid Secretion | 2.82e-002 | 7 | ATP1A1;ATP1A2;CAMK2D;CAMK2G;ITPR1;ITPR2;ITPR3; |
| [hsa04012](http://www.kegg.jp/kegg-bin/show_pathway?hsa04012) | ErbB Signaling Pathway | 1.17e-001 | 6 | AREG;CAMK2D;CAMK2G;MTOR;SRC;STAT5A; |
| [hsa04014](http://www.kegg.jp/kegg-bin/show_pathway?hsa04014) | Ras Signaling Pathway | 8.50e-001 | 6 | CDC42;FGF10;FGFR2;INS;KIT;PDGFB; |
| [hsa04064](http://www.kegg.jp/kegg-bin/show_pathway?hsa04064) | NF-Kappa B Signaling Pathway | 1.38e-001 | 6 | ATM;BCL2;LTA;RIPK1;TNFAIP3;TNFSF11; |
| [hsa04210](http://www.kegg.jp/kegg-bin/show_pathway?hsa04210) | Apoptosis | 1.13e-001 | 6 | ATM;BCL2;PPP3CA;PPP3CB;PPP3R1;RIPK1; |
| [hsa04270](http://www.kegg.jp/kegg-bin/show_pathway?hsa04270) | Vascular Smooth Muscle Contraction | 3.27e-001 | 6 | ADORA2A;ADORA2B;ADRA1B;ITPR1;ITPR2;ITPR3; |
| [hsa04340](http://www.kegg.jp/kegg-bin/show_pathway?hsa04340) | Hedgehog Signaling Pathway | 2.23e-002 | 6 | BMP2;SHH;SMO;WNT10B;WNT4;WNT5A; |
| [hsa04380](http://www.kegg.jp/kegg-bin/show_pathway?hsa04380) | Osteoclast Differentiation | 4.05e-001 | 6 | PPP3CA;PPP3CB;PPP3R1;SPI1;TGFB1;TNFSF11; |
| [hsa04510](http://www.kegg.jp/kegg-bin/show_pathway?hsa04510) | Focal Adhesion | 7.89e-001 | 6 | ACTN3;BCL2;CDC42;CTNNB1;PDGFB;SRC; |
| [hsa04520](http://www.kegg.jp/kegg-bin/show_pathway?hsa04520) | Adherens Junction | 7.00e-002 | 6 | ACTN3;CDC42;CTNNB1;FER;LEF1;SRC; |
| [hsa04726](http://www.kegg.jp/kegg-bin/show_pathway?hsa04726) | Serotonergic Synapse | 2.75e-001 | 6 | CACNA1A;ITPR1;ITPR2;ITPR3;MAOB;SLC6A4; |
| [hsa04914](http://www.kegg.jp/kegg-bin/show_pathway?hsa04914) | Progesterone-Mediated Oocyte Maturation | 1.13e-001 | 6 | HSP90AA1;HSP90AB1;INS;PDE3A;PDE3B;PGR; |
| [hsa04961](http://www.kegg.jp/kegg-bin/show_pathway?hsa04961) | Endocrine And Other Factor-Regulated Calcium Reabsorption | 1.75e-002 | 6 | ATP1A1;ATP1A2;BDKRB2;DNM3;ESR1;VDR; |
| [hsa00030](http://www.kegg.jp/kegg-bin/show_pathway?hsa00030) | Pentose Phosphate Pathway | 1.18e-002 | 5 | G6PD;PGD;PGLS;TALDO1;TKT; |
| [hsa01200](http://www.kegg.jp/kegg-bin/show_pathway?hsa01200) | Carbon Metabolism | 4.24e-001 | 5 | G6PD;PGD;PGLS;TALDO1;TKT; |
| [hsa04141](http://www.kegg.jp/kegg-bin/show_pathway?hsa04141) | Protein Processing In Endoplasmic Reticulum | 7.88e-001 | 5 | BCL2;HSP90AA1;HSP90AB1;STUB1;VCP; |
| [hsa04152](http://www.kegg.jp/kegg-bin/show_pathway?hsa04152) | AMPK Signaling Pathway | 5.43e-001 | 5 | HMGCR;INS;MTOR;SIRT1;SREBF1; |
| [hsa04370](http://www.kegg.jp/kegg-bin/show_pathway?hsa04370) | VEGF Signaling Pathway | 9.82e-002 | 5 | CDC42;PPP3CA;PPP3CB;PPP3R1;SRC; |
| [hsa04650](http://www.kegg.jp/kegg-bin/show_pathway?hsa04650) | Natural Killer Cell Mediated Cytotoxicity | 5.92e-001 | 5 | CSF2;PPP3CA;PPP3CB;PPP3R1;PTK2B; |
| [hsa04668](http://www.kegg.jp/kegg-bin/show_pathway?hsa04668) | TNF Signaling Pathway | 4.47e-001 | 5 | CSF2;EDN1;LTA;RIPK1;TNFAIP3; |
| [hsa04722](http://www.kegg.jp/kegg-bin/show_pathway?hsa04722) | Neurotrophin Signaling Pathway | 5.25e-001 | 5 | BCL2;CAMK2D;CAMK2G;CDC42;YWHAE; |
| [hsa04910](http://www.kegg.jp/kegg-bin/show_pathway?hsa04910) | Insulin Signaling Pathway | 6.50e-001 | 5 | INS;MTOR;PDE3A;PDE3B;SREBF1; |
| [hsa04918](http://www.kegg.jp/kegg-bin/show_pathway?hsa04918) | Thyroid Hormone Synthesis | 1.58e-001 | 5 | ATP1A1;ATP1A2;ITPR1;ITPR2;ITPR3; |
| [hsa04973](http://www.kegg.jp/kegg-bin/show_pathway?hsa04973) | Carbohydrate Digestion And Absorption | 4.02e-002 | 5 | ATP1A1;ATP1A2;GNAT3;TAS1R2;TAS1R3; |
| [hsa04976](http://www.kegg.jp/kegg-bin/show_pathway?hsa04976) | Bile Secretion | 1.58e-001 | 5 | ABCC4;AQP1;ATP1A1;ATP1A2;HMGCR; |
| [hsa00240](http://www.kegg.jp/kegg-bin/show_pathway?hsa00240) | Pyrimidine Metabolism | 6.13e-001 | 4 | NT5C1A;NT5E;POLA1;POLA2; |
| [hsa04070](http://www.kegg.jp/kegg-bin/show_pathway?hsa04070) | Phosphatidylinositol Signaling System | 4.31e-001 | 4 | DGKI;ITPR1;ITPR2;ITPR3; |
| [hsa04120](http://www.kegg.jp/kegg-bin/show_pathway?hsa04120) | Ubiquitin Mediated Proteolysis | 7.89e-001 | 4 | NEDD4;STUB1;UBE2B;UBR5; |
| [hsa04260](http://www.kegg.jp/kegg-bin/show_pathway?hsa04260) | Cardiac Muscle Contraction | 4.15e-001 | 4 | ATP1A1;ATP1A2;RYR2;TPM1; |
| [hsa04360](http://www.kegg.jp/kegg-bin/show_pathway?hsa04360) | Axon Guidance | 7.48e-001 | 4 | CDC42;PPP3CA;PPP3CB;PPP3R1; |
| [hsa04530](http://www.kegg.jp/kegg-bin/show_pathway?hsa04530) | Tight Junction | 7.89e-001 | 4 | ACTN3;CDC42;CTNNB1;SRC; |
| [hsa04611](http://www.kegg.jp/kegg-bin/show_pathway?hsa04611) | Platelet Activation | 7.79e-001 | 4 | ITPR1;ITPR2;ITPR3;SRC; |
| [hsa04630](http://www.kegg.jp/kegg-bin/show_pathway?hsa04630) | Jak-STAT Signaling Pathway | 8.58e-001 | 4 | CSF2;IL10;IL4;STAT5A; |
| [hsa04640](http://www.kegg.jp/kegg-bin/show_pathway?hsa04640) | Hematopoietic Cell Lineage | 4.98e-001 | 4 | CD34;CSF2;IL4;KIT; |
| [hsa04670](http://www.kegg.jp/kegg-bin/show_pathway?hsa04670) | Leukocyte Transendothelial Migration | 6.97e-001 | 4 | ACTN3;CDC42;CTNNB1;PTK2B; |
| [hsa04723](http://www.kegg.jp/kegg-bin/show_pathway?hsa04723) | Retrograde Endocannabinoid Signaling | 5.92e-001 | 4 | CACNA1A;ITPR1;ITPR2;ITPR3; |
| [hsa04744](http://www.kegg.jp/kegg-bin/show_pathway?hsa04744) | Phototransduction | 4.02e-002 | 4 | GNAT1;GNAT3;PDE6A;PDE6B; |
| [hsa04960](http://www.kegg.jp/kegg-bin/show_pathway?hsa04960) | Aldosterone-Regulated Sodium Reabsorption | 8.53e-002 | 4 | ATP1A1;ATP1A2;INS;NR3C2; |
| [hsa04978](http://www.kegg.jp/kegg-bin/show_pathway?hsa04978) | Mineral Absorption | 1.62e-001 | 4 | ATP1A1;ATP1A2;SLC26A6;VDR; |
| [hsa00140](http://www.kegg.jp/kegg-bin/show_pathway?hsa00140) | Steroid Hormone Biosynthesis | 4.64e-001 | 3 | COMT;CYP17A1;CYP19A1; |
| [hsa03030](http://www.kegg.jp/kegg-bin/show_pathway?hsa03030) | DNA Replication | 2.18e-001 | 3 | MCM3;POLA1;POLA2; |
| [hsa03450](http://www.kegg.jp/kegg-bin/show_pathway?hsa03450) | Non-Homologous End-Joining | 2.78e-002 | 3 | LIG4;PRKDC;XRCC4; |
| [hsa04150](http://www.kegg.jp/kegg-bin/show_pathway?hsa04150) | MTOR Signaling Pathway | 4.98e-001 | 3 | HIF1A;INS;MTOR; |
| [hsa04350](http://www.kegg.jp/kegg-bin/show_pathway?hsa04350) | TGF-Beta Signaling Pathway | 6.59e-001 | 3 | BMP2;BMP5;TGFB1; |
| [hsa04610](http://www.kegg.jp/kegg-bin/show_pathway?hsa04610) | Complement And Coagulation Cascades | 5.76e-001 | 3 | BDKRB2;C5;F12; |
| [hsa04621](http://www.kegg.jp/kegg-bin/show_pathway?hsa04621) | NOD-Like Receptor Signaling Pathway | 4.64e-001 | 3 | HSP90AA1;HSP90AB1;TNFAIP3; |
| [hsa04662](http://www.kegg.jp/kegg-bin/show_pathway?hsa04662) | B Cell Receptor Signaling Pathway | 5.92e-001 | 3 | PPP3CA;PPP3CB;PPP3R1; |
| [hsa04672](http://www.kegg.jp/kegg-bin/show_pathway?hsa04672) | Intestinal Immune Network For IgA Production | 3.53e-001 | 3 | IL10;IL4;TGFB1; |
| [hsa04721](http://www.kegg.jp/kegg-bin/show_pathway?hsa04721) | Synaptic Vesicle Cycle | 531e-001 | 3 | CACNA1A;DNM3;SLC18A3; |
| [hsa04727](http://www.kegg.jp/kegg-bin/show_pathway?hsa04727) | GABAergic Synapse | 7.26e-001 | 3 | CACNA1A;HAP1;SRC; |
| [hsa04740](http://www.kegg.jp/kegg-bin/show_pathway?hsa04740) | Olfactory Transduction | 1.00e + 000 | 3 | CAMK2D;CAMK2G;PDE1C; |
| [hsa04913](http://www.kegg.jp/kegg-bin/show_pathway?hsa04913) | Ovarian Steroidogenesis | 4.15e-001 | 3 | CYP17A1;CYP19A1;INS; |
| [hsa04964](http://www.kegg.jp/kegg-bin/show_pathway?hsa04964) | Proximal Tubule Bicarbonate Reclamation | 9.08e-002 | 3 | AQP1;ATP1A1;ATP1A2; |
| [hsa00330](http://www.kegg.jp/kegg-bin/show_pathway?hsa00330) | Arginine And Proline Metabolism | 7.63e-001 | 2 | MAOB;NOS1; |
| [hsa00350](http://www.kegg.jp/kegg-bin/show_pathway?hsa00350) | Tyrosine Metabolism | 5.71e-001 | 2 | COMT;MAOB; |
| [hsa00380](http://www.kegg.jp/kegg-bin/show_pathway?hsa00380) | Tryptophan Metabolism | 5.76e-001 | 2 | IDO1;MAOB; |
| [hsa00480](http://www.kegg.jp/kegg-bin/show_pathway?hsa00480) | Glutathione Metabolism | 6.89e-001 | 2 | G6PD;PGD; |
| [hsa00760](http://www.kegg.jp/kegg-bin/show_pathway?hsa00760) | Nicotinate And Nicotinamide Metabolism | 4.24e-001 | 2 | NT5C1A;NT5E; |
| [hsa00790](http://www.kegg.jp/kegg-bin/show_pathway?hsa00790) | Folate Biosynthesis | 1.62e-001 | 2 | QDPR;SPR; |
| [hsa01230](http://www.kegg.jp/kegg-bin/show_pathway?hsa01230) | Biosynthesis Of Amino Acids | 8.50e-001 | 2 | TALDO1;TKT; |
| [hsa04142](http://www.kegg.jp/kegg-bin/show_pathway?hsa04142) | Lysosome | 9.59e-001 | 2 | CD63;LAMP2; |
| [hsa04145](http://www.kegg.jp/kegg-bin/show_pathway?hsa04145) | Phagosome | 9.88e-001 | 2 | LAMP2;NOS1; |
| [hsa04330](http://www.kegg.jp/kegg-bin/show_pathway?hsa04330) | Notch Signaling Pathway | 6.59e-001 | 2 | HDAC1;HDAC2; |
| [hsa04514](http://www.kegg.jp/kegg-bin/show_pathway?hsa04514) | Cell Adhesion Molecules (CAMs) | 9.88e-001 | 2 | CD34;SELP; |
| [hsa04612](http://www.kegg.jp/kegg-bin/show_pathway?hsa04612) | Antigen Processing And Presentation | 8.50e-001 | 2 | HSP90AA1;HSP90AB1; |
| [hsa04664](http://www.kegg.jp/kegg-bin/show_pathway?hsa04664) | Fc Epsilon RI Signaling Pathway | 8.39e-001 | 2 | CSF2;IL4; |
| [hsa04974](http://www.kegg.jp/kegg-bin/show_pathway?hsa04974) | Protein Digestion And Absorption | 8.81e-001 | 2 | ATP1A1;ATP1A2; |
| [hsa00260](http://www.kegg.jp/kegg-bin/show_pathway?hsa00260) | Glycine, Serine And Threonine Metabolism | 8.62e-001 | 1 | MAOB; |
| [hsa00270](http://www.kegg.jp/kegg-bin/show_pathway?hsa00270) | Cysteine And Methionine Metabolism | 8.58e-001 | 1 | AHCYL1; |
| [hsa00280](http://www.kegg.jp/kegg-bin/show_pathway?hsa00280) | Valine, Leucine And Isoleucine Degradation | 8.81e-001 | 1 | HIBADH; |
| [hsa00340](http://www.kegg.jp/kegg-bin/show_pathway?hsa00340) | Histidine Metabolism | 7.89e-001 | 1 | MAOB; |
| [hsa00360](http://www.kegg.jp/kegg-bin/show_pathway?hsa00360) | Phenylalanine Metabolism | 6.59e-001 | 1 | MAOB; |
| [hsa00561](http://www.kegg.jp/kegg-bin/show_pathway?hsa00561) | Glycerolipid Metabolism | 9.39e-001 | 1 | DGKI; |
| [hsa00564](http://www.kegg.jp/kegg-bin/show_pathway?hsa00564) | Glycerophospholipid Metabolism | 9.88e-001 | 1 | DGKI; |
| [hsa00860](http://www.kegg.jp/kegg-bin/show_pathway?hsa00860) | Porphyrin And Chlorophyll Metabolism | 8.81e-001 | 1 | ALAD; |
| [hsa00900](http://www.kegg.jp/kegg-bin/show_pathway?hsa00900) | Terpenoid Backbone Biosynthesis | 7.04e-001 | 1 | HMGCR; |
| [hsa00982](http://www.kegg.jp/kegg-bin/show_pathway?hsa00982) | Drug Metabolism - Cytochrome P450 | 9.59e-001 | 1 | MAOB; |
| [hsa00983](http://www.kegg.jp/kegg-bin/show_pathway?hsa00983) | Drug Metabolism - Other Enzymes | 8.81e-001 | 1 | HPRT1; |
| [hsa02010](http://www.kegg.jp/kegg-bin/show_pathway?hsa02010) | ABC Transporters | 8.81e-001 | 1 | ABCC4; |
| [hsa03013](http://www.kegg.jp/kegg-bin/show_pathway?hsa03013) | RNA Transport | 1.00e + 000 | 1 | NCBP2; |
| [hsa03015](http://www.kegg.jp/kegg-bin/show_pathway?hsa03015) | MRNA Surveillance Pathway | 9.88e-001 | 1 | NCBP2; |
| [hsa03040](http://www.kegg.jp/kegg-bin/show_pathway?hsa03040) | Spliceosome | 1.00e + 000 | 1 | NCBP2; |
| [hsa03410](http://www.kegg.jp/kegg-bin/show_pathway?hsa03410) | Base Excision Repair | 8.50e-001 | 1 | POLB; |
| [hsa03420](http://www.kegg.jp/kegg-bin/show_pathway?hsa03420) | Nucleotide Excision Repair | 8.81e-001 | 1 | CETN2; |
| [hsa04115](http://www.kegg.jp/kegg-bin/show_pathway?hsa04115) | P53 Signaling Pathway | 9.59e-001 | 1 | ATM; |
| [hsa04130](http://www.kegg.jp/kegg-bin/show_pathway?hsa04130) | SNARE Interactions In Vesicular Transport | 8.50e-001 | 1 | VTI1A; |
| [hsa04140](http://www.kegg.jp/kegg-bin/show_pathway?hsa04140) | Regulation Of Autophagy | 8.62e-001 | 1 | INS; |
| [hsa04614](http://www.kegg.jp/kegg-bin/show_pathway?hsa04614) | Renin-Angiotensin System | 6.50e-001 | 1 | AGT; |
| [hsa04620](http://www.kegg.jp/kegg-bin/show_pathway?hsa04620) | Toll-Like Receptor Signaling Pathway | 1.00e + 000 | 1 | RIPK1; |
| [hsa04622](http://www.kegg.jp/kegg-bin/show_pathway?hsa04622) | RIG-I-Like Receptor Signaling Pathway | 9.59e-001 | 1 | RIPK1; |
| [hsa04623](http://www.kegg.jp/kegg-bin/show_pathway?hsa04623) | Cytosolic DNA-Sensing Pathway | 9.59e-001 | 1 | RIPK1; |
| [hsa04666](http://www.kegg.jp/kegg-bin/show_pathway?hsa04666) | Fc Gamma R-Mediated Phagocytosis | 9.88e-001 | 1 | CDC42; |
| [hsa04920](http://www.kegg.jp/kegg-bin/show_pathway?hsa04920) | Adipocytokine Signaling Pathway | 9.59e-001 | 1 | MTOR; |
| [hsa04962](http://www.kegg.jp/kegg-bin/show_pathway?hsa04962) | Vasopressin-Regulated Water Reabsorption | 8.81e-001 | 1 | AVPR2; |

*Annotation:“Targets” are referred to as the targets mapped to this term.

**Supplementary table 5. Chart of the docking simulation with docking scores (pKd/pKi) of these 5 targets with specified proteins.**

| **No.** | **Origianl Protein Name** | **User-specified Protein Name** | **PDB ID** | **Test Compounds** | **Docking Scores (pKd/pKi)** |
| --- | --- | --- | --- | --- | --- |
| 1 | ESR1 | ESR1 | 3CBP | 636458 | 5.769 |
| 2 | ESR1 | ESR1 | 3CBP | 6436348 | 6.021 |
| 3 | ESR1 | ESR1 | 3CBP | 6441391 | 5.815 |
| 4 | ESR2 | ESR2 | 1QKM | 636458 | 6.883 |
| 5 | ESR2 | ESR2 | 1QKM | 6436348 | 7.349 |
| 6 | ESR2 | ESR2 | 1QKM | 6441391 | 7.376 |
| 7 | TP53(P53) | TP53(P53) | 3D06 | 636458 | 6.02 |
| 8 | TP53(P53) | TP53(P53) | 3D06 | 6436348 | 5.94 |
| 9 | TP53(P53) | TP53(P53) | 3D06 | 6441391 | 5.209 |
| 10 | SRC | SRC | 1O4R | 636458 | 5.866 |
| 11 | SRC | SRC | 1O4R | 6436348 | 5.951 |
| 12 | SRC | SRC | 1O4R | 6441391 | 4.69 |
| 13 | VDR | VDR | 1IE9 | 636458 | 6.157 |
| 14 | VDR | VDR | 1IE9 | 6436348 | 5.96 |
| 15 | VDR | VDR | 1IE9 | 6441391 | 7.143 |

**Supplementary Table 6.** Summary of the enriched categories of diseases in Disgenet. This table lists the enriched categories, number of targeted genes in the user gene list and also in the categories and FDR. Categories with red color represent the positive related categories while categories with blue color represent the positive related categories.

| **ID** | **Name** | **#Gene** | **Gene Symbol** | **FDR** | **Pvalue** |
| --- | --- | --- | --- | --- | --- |
| umls:C1458155 | Mammary Neoplasms | 11 | ALL | 7.81e-01 | 0e+00 |
| umls:C0001418 | Adenocarcinoma | 7 | CYP19A1,ESR1,ESR2,PGR,PIK3CA | 6.03e-01 | 2.68e-01 |
| umls:C0033578 | Prostatic Neoplasms | 7 | CYP19A1,ESR1,ESR2,ATM,PIK3CA | 1e+00 | 2.62e-01 |
| [umls:C0005695](#umls:C0005695) | Bladder Neoplasm | 5 | ESR1,ATM | 6.15e-01 | 4.04e-01 |
| [umls:C2239176](#umls:C2239176) | Liver carcinoma | 5 | ESR1,ATM,PIK3CA | 6.86e-01 | 5.12e-01 |

**Supplementary Table Table 7.** Target genes/proteins, pathways and ingredients of Traditional Medicines combined with breast cancer that searched by the keyword in original papers in Pubmed and clinical trials from clinicaltrials database that can prove their roles.

| **Targets** | **Keywords** | **Num of Papers** | **Clinical Trials** | |
| --- | --- | --- | --- | --- |
|  |  |  | **Num** | **Principal Sponsors** |
| **11 Target genes/proteins** | | | | |
| ESR1 | “ESR1” AND "breast cancer" | 541 | 846 | United States (472), Europe (275), China (103), Canada (88), South America (50), Austrilia (44), Middle East (37), North Asia (35), Southeast Asia (29), Japan (28), Africa (25), Mexico (20), South Asia (15). |
| ESR2 | “ESR2” AND "breast cancer" | 65 | 10 | United States (5 ), China (2), France (2), Israel (1), Swiss (1), Poland (1). |
| TP53 | “TP53” AND "breast cancer" | 1392 | 16 | United States (10), Europe (6) |
| SRC | “SRC” AND "breast cancer" | 1342 | 11 | United States(7), Europe(4), Canada(1), North Asia(1) |
| VDR | “VDR” AND "breast cancer" | 269 | 6 | United States(4), South Asia(1),Africa (1) |
| PIK3CA | “PIK3CA” AND "breast cancer" | 548 | 35 | Europe(25), United States(19), China (11), Southeast Asia (10), Canada (9), Pacifica (8), South America (7), Middle East (5), Mexico (4), Japan (4), South Asia (1),Africa (1),Central America(1) |
| PGR | “PGR” AND "breast cancer" | 1634 | 297 | United States(203), Europe(71), Canada(28), Pacifica(15),South America(15),China (14), Africa (10), Central America (13),Japan(4),Middle East(11),Mexico(6),North Asia(5),South Asia(4),Southeast Asia(6) |
| ATM | “ATM” AND "breast cancer" | 650 | 11 | United States(8), Europe(4), China (1) |
| CYP19A1 | “CYP19A1” AND "breast cancer" | 122 | 706 | United States(359), Europe(255), China(114), Canada(75), South America(44), Pacifica(40), Middle East(34), North Asia(34), Japan(28),Southeast Asia(28), Africa(22), Mexico(18),Central America(17), South Asia(17) |
| HSP90AA1 | “HSP90AA1” AND "breast cancer" | 4 | 1 | China(1,Protgen Ltd) |
| PHB | “PHB” AND "breast cancer" | 20 | 0 | - |
| **4 key pathways** | | | | |
| Estrogen signaling pathway | “Estrogen signaling pathway” AND "breast cancer" | 43 | 27 | United States(18), Europe(5), China(1), Canada(1), South America(1), Pacifica(1) |
| Prolactin signaling pathway | “Prolactin signaling pathway” AND "breast cancer" | 1 | 0 | - |
| Progesterone-Mediated Oocyte Maturation | “Progesterone-Mediated Oocyte Maturation” AND "breast cancer" | 1 | 0 | - |
| Thyroid Hormone Signaling Pathway | “Thyroid Hormone Signaling Pathway” AND "breast cancer" | 0 | 0 | - |
| **3 ingredients** | | | | |
| germacrone | "germacrone" AND "breast cancer" | 6 | 0 | - |
| curdione | "curdione" AND "breast cancer" | 2 | 0 | - |
| furanodiene | "furanodiene" AND "breast cancer" | 9 | 0 | - |

#### Note: Published Papers were searched from PubMed (https://www.ncbi.nlm.nih.gov/pubmed), Clinical Trials were searched from clinicaltrials (https://clinicaltrials.gov). Data current was April 10, 2017.
